# AMPK γ-Subunit Isoform Switching Governs Temozolomide Resistance and Survival in Glioblastoma

**DOI:** 10.3390/cells15171594

**Published:** 2026-09-02

**Authors:** Shweta Dongre, Arpit Sharma, Naveen Soni, Bhawana Bissa

**Affiliations:** Department of Biochemistry, Central University of Rajasthan, Ajmer 305817, India; 2023phdbc004@curaj.ac.in (S.D.); as95319286@gmail.com (A.S.); 2021phdbc005@curaj.ac.in (N.S.)

**Keywords:** AMPK subunit, glioblastoma (GBM), autophagy, temozolomide, chemoresistance, cancer metabolism

## Abstract

**Highlights:**

**What are the main findings?**

- TMZ treatment induces a dynamic switch in AMPK γ-subunit composition from γ1 to γ2 and enhances AMPK activation in GBM cells.
- AMPKγ2 promotes TMZ resistance because its overexpression increases survival and AMPK activation, whereas its silencing sensitizes GBM cells to TMZ.

**What are the implications of the main findings?**

- The γ1 to γ2 switch represents a novel mechanism of metabolic adaptation that enables GBM cells to survive chemotherapy-induced energetic stress.
- Targeting AMPKγ2 or preventing AMPK isoform switching may provide a promising therapeutic strategy to overcome TMZ resistance and improve glioblastoma treatment outcomes.

**Abstract:**

Temozolomide (TMZ) resistance remains a fundamental obstacle in the treatment of glioblastoma (GBM). While the metabolic sensor AMPK is known to influence cancer cell survival, the specific role of its regulatory γ-subunit isoforms in orchestrating chemoresistance is poorly understood. This study investigates how the dynamic remodeling of the AMPK heterotrimer contributes to TMZ evasion in GBM. We observed significantly low expression of AMPKγ2 in glioma patient samples, but the treatment of GBM cell lines with TMZ led to a robust increase in AMPKγ2 expression with a concomitant decrease in AMPKγ1 expression. We identified a significant “isoform switch” in TMZ-treated cells, characterized by a marked downregulation of the γ1 subunit and a reciprocal upregulation of γ2. The structural remodeling of the AMPK complex was validated using co-immunoprecipitation (Co-IP). Co-IP analysis confirmed that the AMPK α catalytic subunit shifts its primary association from γ1 to γ2 during the TMZ treatment. Functionally, γ2-dominant complexes exhibited reduced sensitivity to ATP-mediated inhibition, allowing resistant cells to maintain better ATP homeostasis and sustained AMPK activation under TMZ-induced stress. Furthermore, the knockdown of the γ2 subunit abolished this metabolic advantage, resulting in the resensitization of GBM cell lines to TMZ-induced cell death. Our findings reveal that AMPK γ-subunit isoform switching is a previously unrecognized metabolic adaptation that drives TMZ resistance in GBM. Targeting the γ2-specific complex or preventing this isoform transition represents a promising therapeutic strategy to overcome chemoresistance in malignant gliomas.

## 1. Introduction

Cellular survival depends on the ability to sense and adapt to fluctuating energy demands across eukaryotes. AMP-activated protein kinase (AMPK) functions as a key regulator of this process [1,2]. It is a conserved serine/threonine kinase that functions as a molecular energy sensor. AMPK acts as a molecular switch by monitoring adenine nucleotide levels and activating catabolic pathways to restore ATP while suppressing anabolic processes [3]. Although initially it was characterized as a cell-autonomous fuel gauge in multicellular organisms, it has evolved to integrate nutrient and hormonal signals and hence coordinate the energy balance across tissues [4]. AMPK functions as a heterotrimeric complex composed of a catalytic α subunit, a scaffold β subunit that connects the α and γ subunits together, and a regulatory γ subunit [5]. Each subunit exists in multiple isoforms, α1/α2 (encoded by *PRKAA1* and *PRKAA2*), β1/β2 (*PRKAB1, PRKAB2*), and γ1/γ2/γ3 (*PRKAG1, PRKAG2, PRKAG3*) [6], giving rise to a variety of functional AMPK complexes in human cells [7]. AMPK regulates numerous metabolic processes, including glucose and lipid metabolism, autophagy, and mitochondrial biogenesis, by phosphorylating downstream targets [8]. AMPK activation is primarily mediated by phosphorylation at T172 by upstream kinases such as LKB1 [9] and CaMKKβ in response to cellular energy stress [10].

Glioblastoma (GBM), classified as an IDH-wildtype grade IV tumor according to the 2021 WHO classification of tumors of the central nervous system, is the most aggressive and primary malignant brain tumor in adults [11]. It originates within the brain rather than arising from the metastatic spread of tumors from other organs [12]. GBM accounts for nearly 60% of the adult brain tumors and is characterized by rapid proliferation, extensive heterogeneity, metabolic reprogramming, and poor clinical prognosis [13]. Despite advances in surgical resection, radiotherapy, and chemotherapy, the median survival of GBM patients remains limited. In GBM treatment, the development of chemoresistance is a major challenge, including resistance to temozolomide (TMZ), a standard chemotherapeutic drug used in GBM therapy [14]. Tumor cells often adapt to metabolic and therapeutic stress by activating survival pathways that promote proliferation and resistance to apoptosis [15]. In this context, AMPK signaling has been shown to promote adaptive survival mechanisms, including autophagy and metabolic reprogramming, in response to chemotherapeutic stress [16,17].

Increasing evidence suggests that metabolic regulators play a crucial role in mediating this adaptive response. Recent studies have implicated AMPK signaling in cancer progression and therapy response, including glioblastoma [18,19]. While AMPK has traditionally been described as a tumor suppressor due to its role in inhibiting anabolic growth pathways, cancer cells can exploit AMPK signaling to survive under metabolic stress conditions induced by hypoxia, nutrient limitations, or chemotherapy [20]. The regulatory γ subunit of AMPK is particularly important because it senses cellular energy status through the binding of AMP, ADP, and ATP, thereby controlling AMPK activation [21]. However, the isoform-specific contribution of AMPK γ subunits in glioblastoma remains poorly understood. In particular, differential expression or functional switching between the γ1 and γ2 isoforms may alter AMPK signaling dynamics and influence cellular adaptation to chemotherapeutic stress [22]. Given that γ subunits determine nucleotide sensitivity and the activation dynamics of AMPK [23], isoform-specific switching may represent a critical but underexplored mechanism of metabolic adaptation in glioblastoma. In this study, we investigate the expression and functional significance of AMPK γ subunits in glioblastoma, revealing a potential γ-subunit isoform switching phenomenon associated with temozolomide response. Understanding this mechanism may provide new insight into AMPK-mediated metabolic adaptation and chemoresistance in GBM.

## 2. Material and Methods

### 2.1. GBM Sample Collection

GBM tumor tissues (Table 1) were obtained from surgical resection from the Department of Neurosurgery at All India Institute of Medical Sciences, Delhi, India. All samples were collected with the patients’ consent and in accordance with the protocol approved by the Institutional Ethics Committee Central University of Rajasthan (CURAJ/IEC/2020/SNO02 on 3 July 2020) for subsequent PCR analysis.

### 2.2. In Silico Analysis

Clinical data were obtained from public databases to understand the expression of AMPK genes. Databases including GEPIA [24], GlioVis, and the Human Protein Atlas (HPA) [25] were analyzed for the expression analysis. GlioVis is a user-friendly database for the visualization and analysis of brain tumor expression datasets. It offers an interactive interface to analyze gene expression profiles across brain cancer and normal tissues, survival analysis, correlation analysis between gene expressions, etc. We analyzed RNA sequencing data from brain cancer/normal tissues in The Cancer Genome Atlas (TCGA) and the Clinical Proteomic Tumor Analysis Consortium (CPTAC) to assess protein expression. HPA was used to get IHC images. For the survival plot, GEPIA was used.

### 2.3. Molecular Docking Simulations

Molecular docking simulations were conducted to evaluate the binding interaction between the ligand, temozolomide, ATP, and the γ1 subunit of AMPK.

#### 2.3.1. Ligand Preparation

The three-dimensional structure of temozolomide (PubChem CID: 5394) and ATP (PubChem CID: 5486828) was retrieved from the NCBI PubChem database [26] in Structure Data Format (SDF). The ligand file was subsequently converted to the Protein Data Bank (PDB) format using Open Babel [27]. During this conversion, a weighted rotor search was applied to generate the lowest energy conformer. The final ligand preparation for docking, including the addition of polar hydrogens, calculation of Gasteiger charges, and definition of rotatable bonds via root selection, was performed using Raccoon. The processed ligand was saved in the Protein Data Bank, Partial Charge (Q), and Atom Type (T) (PDBQT) format.

#### 2.3.2. Receptor Preparation

The three-dimensional crystal structure of AMPK was obtained from the RCSB Protein Data Bank [28] with the PDB ID 4QFR. To isolate the γ1 subunit for targeted docking, the alpha and beta subunits were removed, leaving only the γ1 chain. The remaining γ1 structure was further prepared by removing all heteroatoms, including water molecules, cofactors, and any bound ligands, using BIOVIA Discovery Studio Visualizer v25.1.0.24284 [29] and PyMOL 3.1 [30]. The isolated γ1 subunit was then subjected to energy minimization using the Swiss PDB Viewer [31] with the GROMOS-14B1 force field. For docking preparation, polar hydrogen atoms were added, and Kollman charges were assigned (resulting in partial charges of −8.376 for α-A and −6.248 for α-G) using AutoDock Tools v1.5.7 [32]. The fully prepared γ1 receptor was saved in PDBQT format.

#### 2.3.3. Docking Protocol and Analysis

Molecular docking was performed using the CB-Dock web server 3 [33], an automated protein–ligand docking tool that employs curvature-based cavity detection for binding site prediction. The server identifies potential binding cavities on the γ1 subunit, calculates their centers and sizes, and customizes the docking box dimensions accordingly. The actual docking calculation was executed by AutoDock Vina 1.5.7 [34]. Following the simulation, the resulting binding poses were analyzed. The most favorable binding configuration was selected based on the highest AutoDock Vina binding affinity score (kcal/mol) and the appropriateness of the cavity volume for temozolomide and visualized in discovery studio 2025 client [35].

### 2.4. Cell Culture and Reagents

Human glioblastoma cells (LN229 and U87MG) were procured from the National Centre for Cell Sciences (NCCS), Pune, India. The cells were cultured in Dulbecco’s Modified Eagle Medium (DMEM) (11965-092, Gibco, Thermo Fisher Scientific, Waltham, MA, USA), supplemented with 10% Fetal Bovine Serum (FBS) (A5256701 Gibco) and 1% antibiotic (streptomycin and penicillin) in a humidified atmosphere of 5% CO_2_ at 37 °C and were sub-cultured twice a week. Temozolomide (TMZ) (100 mM stock solution) was dissolved in dimethyl sulfoxide (DMSO) (TC185, HI media) and stored at −20 °C.

### 2.5. RNA Isolation and PCR

One microgram of total RNA (isolated using the TRIzol method) was used for first-strand cDNA synthesis with the cDNA synthesis kit (Verso, Thermo Fisher). A PCR was performed using the EmeraldAmp PCR Master Mix (#RR310A, Takara, Kusatsu, Shiga, Japan) and Eurofins Genomics primer (Bengaluru, Karnataka, India) in (Table 2) a Veriti 96-well thermal cycler (Applied Biosystems, Thermo Fisher Scientific). Samples were run in agarose gel electrophoresis, and gel image analysis and quantification were performed using ImageJ 1.8.0. GraphPad Prism 8.0.1 was used for graph preparation and statistical analysis.

### 2.6. Plasmid Transfection and shRNA

Plasmids pWZL Neo Myr Flag *PRKAG2* (Addgene ID: 20656), pECE-M2-AMPKα2 wt (Addgene ID: 31652), AMPKβ1 Flag (Addgene ID: 40602), AMPKγ1 HA (Addgene ID: 40605), and AMPKβ2 MYC (Addgene ID: 40606) were used to overexpress the gene. Flag AMPKα1 was obtained from Dr. Jingyue Jia, Assistant Professor, Department of Internal Medicine, University of New Mexico, Albuquerque, NM, USA. The shAMPKγ2 plasmid (Clone ID: TRNC0000003148; Target seq—GAAGTGCAATAAGCTGGAAAT; Gene ID: 51422; Ref Seq: NM_016203) was used for the knockdown experiments. Cells were transfected using the Xfect Transfection Reagent (Takara #631317) according to the manufacturer’s protocol.

### 2.7. Cell Viability (MTT) Assay and Crystal Violet

LN229 and U87MG cells were grown in complete DMEM in a 96-well plate to reach ~70% confluency. Different concentrations of temozolomide (100 μM, 200 μM, and 400 μM) were used for 24 h treatment. To measure cell viability, the 3-(4,5-dimethylthiazol-2-yl)-2,5-diphenyl-tetrazolium bromide (MTT) assay was used. After the completion of the treatment, 10 μL of MTT (5 mg/mL) was added to each well and incubated at 37 °C. After 2–3 h of incubation, the medium with MTT was discarded, and 100 μL of DMSO was added to each vial to suspend the crystal. Absorbance readings were obtained using a microplate reader at 570 nm. IC50 was calculated using the OD readings. For imaging, 0.5% crystal violet stain was used. After PBS washing, images were taken using brightfield and phase-contrast microscopy.

### 2.8. Antibodies

The antibodies were purchased from either Cell Signaling Technology (CST) or Proteintech. Antibodies against AMPKα1 Mouse mAb (66536-1-Ig), AMPKα2 Rabbit pAb (18167-1-AP), AMPKβ1 (71C10) Rabbit mAb (#4178), AMPKβ2 Rabbit pAb (#4148), AMPKγ1 Rabbit pAb (#4187), AMPKγ2 Rabbit pAb (#2536), pAMPK alpha (T172) (40H9) Rabbit mAb (#2535T), LC3A/B (D3U4C) XP (R) Rabbit mAb (#1271T), Beta-actin Mouse mAb (8H10D10), PARP (#46D11), P62/SQSTM1 (#MAB8028), Anti-mouse IgG HRP-linked antibody (7076P2), and Anti-rabbit IgG HRP-linked antibody (#7074) were used.

### 2.9. Whole Cell Lysate Preparation and Western Blotting

Cell lysate was prepared from 60–100 mm culture dishes. Cells were washed with phosphate-buffered saline (PBS) and lysed with NP-40 lysis buffer (25 mM Tris pH 8, 150 Mm NaCl, 0.1% NP-40, 0.1 mM EDTA), and 1×protease inhibitor cocktail (PIC) (ML051, Himedia, Kennett Square, PA, USA) was added. The lysate was incubated on ice for 20–25 min with vortexing every 5 min. A clear supernatant was collected by centrifugation at 12,000 rpm for 15 min at 4 °C. Protein concentration was determined using Bicinchoninic Acid (BCA, Thermo Fisher) reagents. For SDS-PAGE, 100 μg of protein lysate was loaded onto a gel and separated under denaturing conditions. The separated proteins were then transferred onto a Bio-Rad immunoblot PDVF membrane (charged with methanol) using the wet transfer method. The membrane was blocked for 1 h with 3% BSA (blocking buffer) on a rocker at room temperature. It was then incubated with primary antibody (1:1000 dilution in 3% BSA) overnight at 4 °C. Afterward, the membrane was gently washed three times with TBST (TBS buffer with 0.1% Tween-20) and incubated with an HRP-tagged secondary antibody (1:3000) for 2 h. Following three very gentle TBST washes, the blot was visualized using a blot scanner.

### 2.10. Co-Immunoprecipitation (Co-IP)

A total of 250 μg of total protein lysate was incubated overnight at 4 °C with anti-FLAG antibody along with continuous rocking. Immune complexes were captured using Dynabeads Protein G and washed twice with NP-40 lysis buffer to remove non-specifically bound proteins. A 5× SDS sample buffer was added to the bead-bound complexes, which were then heated to elute the bound protein. The samples were subsequently processed for Western blot analysis. Input lysate and immunoprecipitated fractions were analyzed.

### 2.11. JC-1 Mitochondrial Membrane Integrity Analysis

JC-1 dye (5,5′,6,6′-tetrachloro-1,1′,3,3′-tetraethybenimidazolylcarbocyanine iodide) (Thermo Fisher Scientific #T3168) was dissolved in DMSO to prepare a stock solution of 100 mM. Phosphate-buffered saline (PBS) was used for the washing steps. Cells were harvested after 24 h of transfection and 24 h of TMZ treatment and washed twice with PBS. JC-1 dye was added at a final working concentration of 0.3 mM, and cells were incubated in the dark at 37 °C for 20 min. Following incubation, cells were washed twice with PBS to remove excess dye. Fluorescence was measured using a fluorescence microscope.

### 2.12. AMPKα (pT172) Kinase Activity Assay

AMPK activity was determined by measuring the phosphorylation of AMPKα at T172 using an AMPKα (pT172) ELISA kit (Thermo Fisher Scientific #KHO0651), according to the manual’s instructions. Cells from different treatment conditions were lysed in the provided buffer containing protease and phosphatase inhibitors, and clarified lysates were collected after centrifugation. An equal amount of protein was added to the ELISA wells pre-coated with phospho-specific AMPKα (Thr 172) antibody and incubated, followed by detection using secondary antibody. Absorbance was measured at 450 nm.

### 2.13. APO BrdU TUNEL Assay

U87MG cells were seeded in 60 mm dishes with a density of 3 × 10^4^ cells per dish. At 60% confluence, the cells were treated with shAMPKγ2 for 24 h and then treated with TMZ (IC50). Cells were fixed with 1% paraformaldehyde. TUNEL assay, which is used to detect DNA damage, was conducted using an APO-BrdU TUNEL assay kit (#A23210, Invitrogen, Thermo Fisher Scientific), as per the manufacturer’s protocol. Hoechst stain was used as a counterstain for the nucleus. The cells were analyzed under confocal microscopy (Leica, Stellaris 8 Wetzlar, Germany). A higher level of green fluorescence in the nucleus means the cells are actively undergoing apoptosis, while red fluorescence (propidium iodide) serves as a counterstain for the cell cycle and identifies necrotic cells.

### 2.14. Statistical Analysis

All data analyses were done using GraphPad Prism 8.0. We used multiple tests, such as a One-way ANOVA, Two-way ANOVA, and *t*-tests, depending on variables, to estimate the difference between groups. A *p*-value of <0.05 was considered significant in the whole study (* = *p* < 0.05; ** = *p* < 0.01; *** = *p* < 0.001; **** = *p* < 0.0001). The densitometric calculation was done using ImageJ.

## 3. Results

### 3.1. Differential Expression of AMPK Subunits in GBM

**Figure 1:**
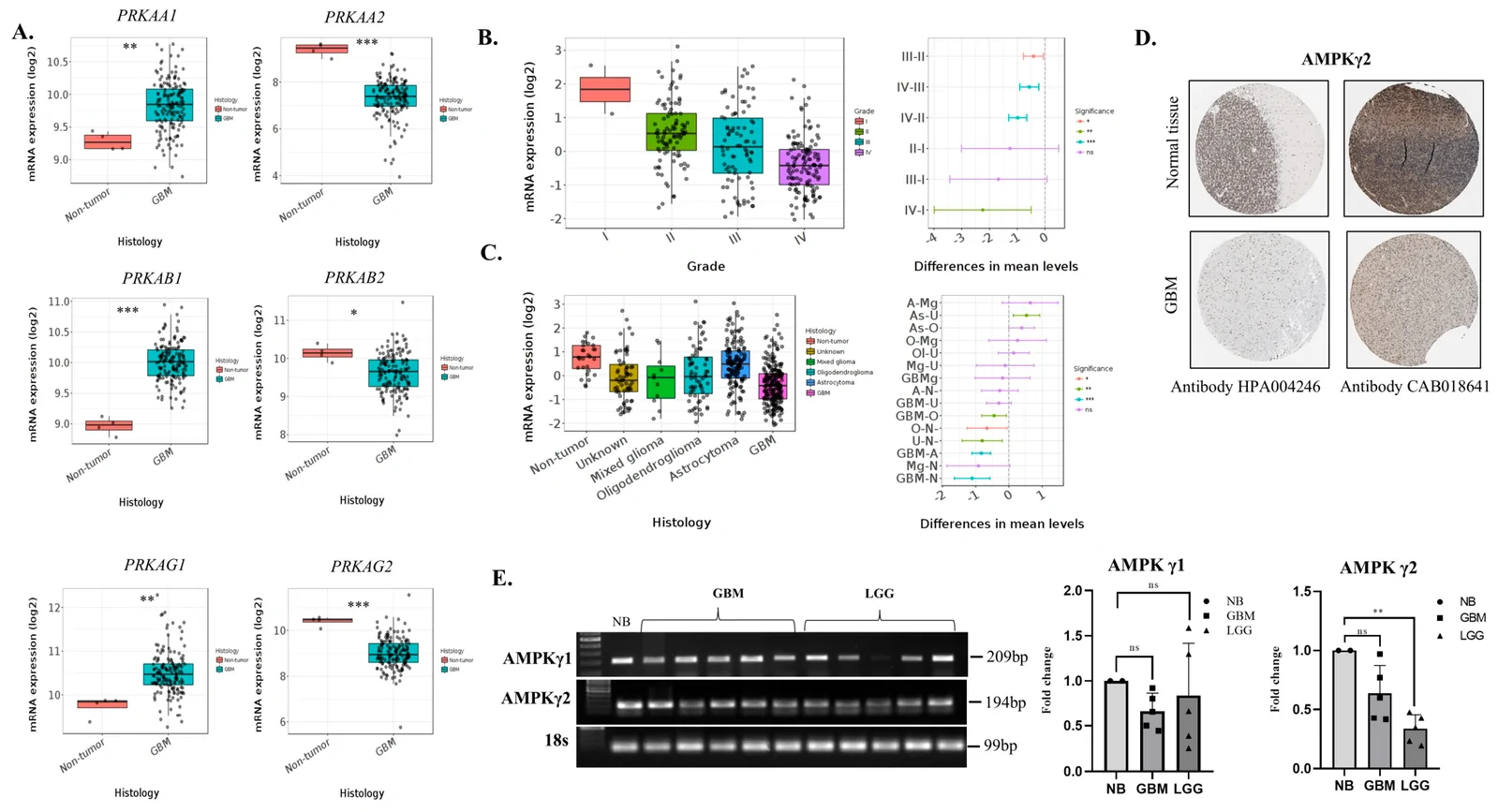
Differential expression of AMPK subunits in GBM. (**A**) Expression analysis of AMPK subunit genes *PRKAA1*, *PRKAA2*, *PRKAB1*, *PRKAB2*, *PRKAG1*, and *PRKAG2* in glioblastoma (GBM). * *p* < 0.05, ** *p* < 0.01, *** *p* < 0.001. (**B**,**C**) mRNA expression analysis of *PRKAG2* across different glioma tumor grades and histological subtypes. (**D**) Immunohistochemistry (IHC) images showing AMPKγ2 expression in normal brain tissue and GBM using HPA004246 and CAB018641 antibody. (**E**) RT-PCR validation of AMPKγ1 and AMPKγ2 expression. Data are presented as mean ± SD. ** *p* < 0.01, ns = not significant.

The transcriptional landscape of AMPK subunits in glioma was assessed using the web server GlioVis 0.2.0 [36]. It integrates multiple large-scale brain tumor datasets, including TCGA RNA-seq profiles, and enables the standardized visualization of gene expression across tumor subtypes and clinical annotations. Using the database, we examined the mRNA expression patterns of AMPKα (*PRKAA1/2*), AMPKβ (*PRKAB1/2*), and AMPKγ (*PRKAG1/2*) subunits across glioma samples. We first assessed AMPK subunit expression in non-tumor and GBM samples (Figure 1A) and observed clear subtype-specific differences. *PRKAA1*, *PRKAB1*, and *PRKAG1* were significantly highly expressed, whereas the expression of *PRKAA2*, *PRKAB2*, and *PRKAG2* was significantly low in GBM compared to the non-tumor sample. When the datasets were further stratified by WHO tumor grade (I–IV) classification (Figure 1B), a similar divergence emerged. These observations align with earlier transcriptomic studies, which have shown that AMPK components may undergo independent transcriptional regulation in cancer [37], with the selective enrichment in or suppression of individual subunits contributing to metabolic rewiring and tumor progression. A significant decrease in *PRKAG2* expression was also observed across histological subtypes, including oligodendroglioma, astrocytoma, and GBM (Figure 1C). Although AMPK operates as a heterotrimeric kinase complex, the individual subunits displayed distinct, non-uniform expression profiles. Notably, AMPKγ2 (*PRKAG2*) showed a consistent and significant decrease in GBM compared to non-tumor brain tissue as well as lower-grade gliomas in both histology-based and grade-based analyses. This suppression appeared to be specific to γ2 and was not uniformly observed for γ1. The immunohistochemistry analysis of AMPKγ2 expression from the Human Protein Atlas (HPA) represents a clear difference between normal brain tissue and GBM (Figure 1D). AMPKγ2 expression (antibody HPA0042446 and CAB018641) decreases in GBM compared to normal brain tissue.

Supporting these data, the correlation analysis of AMPK subunits demonstrates distinct association patterns, suggesting coordinated yet selective regulation within AMPK complexes (Supplementary Figure S1A). PRKAG2 expression was observed to be significantly lower in GBM compared to the normal brain, determined by transcript-level validation using TCGA datasets (Supplementary Figure S1B). This reduction was constant across patient age groups and did not show a discernible age-dependent trend (Supplementary Figure S1C). To investigate potential mechanisms underlying PRKAG2 downregulation, promoter methylation analysis revealed increased methylation levels in GBM tumors compared to normal samples (Supplementary Figure S1D), while age-stratified analysis showed no consistent methylation pattern across groups (Supplementary Figure S1E). Interestingly, CpG site-specific analysis revealed no statistically significant difference for PRKAG1, whereas PRKAG2 showed a trend towards increased methylation in tumor samples (Supplementary Figure S1G). Consistent with transcriptomic data, proteomic analysis from CPTAC datasets demonstrated reduced PRKAG2 protein levels in GBM tumors relative to normal tissue (Supplementary Figure S1F), confirming downregulation at multiple levels. The γ subunit is critical because it governs AMP/ATP sensing and thereby dictates AMPK activation in response to metabolic stress [16]. To determine whether γ-subunit regulation affects tumor inhibition or supports tumor survival, we examined the expression patterns of AMPKγ1 and AMPKγ2 in glioma patient samples. An analysis of GBM and LGG patient samples revealed distinct expression differences between the two isoforms. Real-time PCR revealed that while AMPKγ1 was consistently expressed across all groups, AMPKγ2 displayed notably reduced expression in both GBM and LGG (low-grade glioma) compared to the NB (normal brain) (Figure 1E). This selective loss of γ2 suggests the attenuation of a potentially stress-responsive AMPK function during glioma progression.

### 3.2. AMPK Subunit Expression Is Altered Under TMZ Treatment

**Figure 2:**
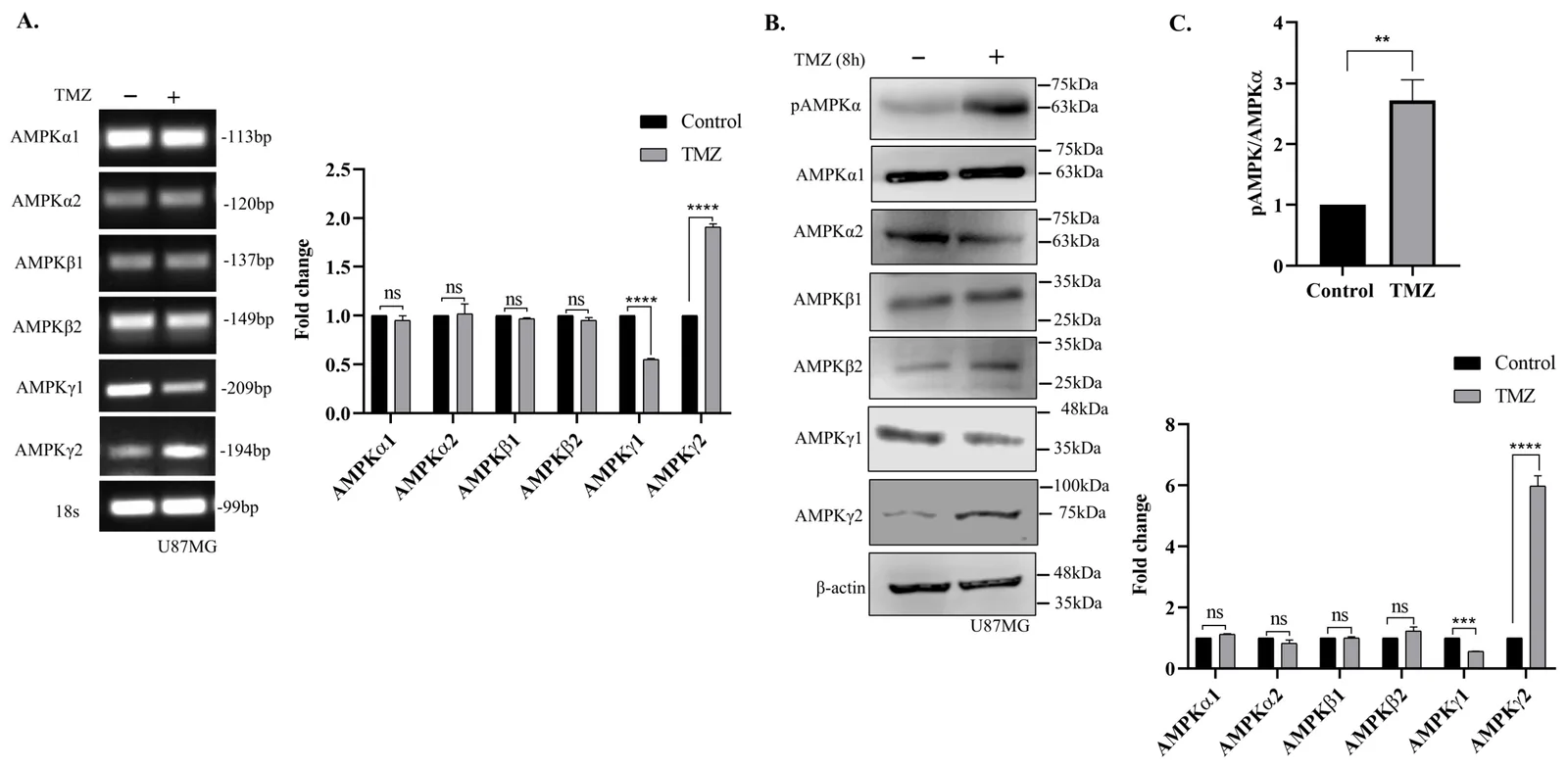
AMPK γ subunits show differential expression in response to TMZ treatment. (**A**) Semi-quantitative and real-time PCR analysis of AMPK subunit transcripts in U87MG cells following temozolomide (TMZ) treatment. Representative gel images with expected amplicon sizes are shown. Bar graph represents fold change in mRNA expression level relative to untreated control, normalized with 18S rRNA. (**B**) Western blot analysis of AMPK subunits in U87MG cells treated with TMZ for 8 h. Protein levels of pAMPK, AMPKα1, AMPKα2, AMPKβ1, AMPKβ2, AMPKγ1, and AMPKγ2 were examined, with β-actin used as loading control. (**C**) Densitometric quantification of Western blot bands showing fold changes in protein expression relative to control. pAMPK/AMPK ratio indicated increased AMPK activation upon TMZ treatment. Notably, AMPKγ1 expression decreases, whereas AMPKγ2 expression increases. Data are presented as mean ± SD of three biological repeats. Statistical significance was determined relative to control (** *p* < 0.01, *** *p* < 0.001, **** *p* < 0.0001; ns—not significant).

To determine whether AMPK subunit expression is altered under therapeutic stress, we examined their expression in GBM cell lines, U87MG and LN229, following treatment with temozolomide (TMZ). TMZ is a DNA-alkylating agent and the current clinical standard for GBM therapy. It is known to activate AMPK signaling and induce metabolic stress responses in glioblastoma cells [38]. To further examine the kinetics of AMPK activation, U87MG cells were treated with TMZ (358 μM) and harvested at different time points (0, 4, 8, and 12 h). Western blot analysis revealed that phosphorylated AMPK (pAMPK) levels increased over time, and densitometric analysis was performed by plotting the pAMPK/AMPK ratio, which confirmed the maximal activation at the 8 h time point (Supplementary Figure S2A). This observation is consistent with previous reports demonstrating the time-dependent activation of AMPK upon TMZ treatment, with the maximal phosphorylation observed around 8 h in U87MG cells [39]. U87MG and LN229 cells were divided into two groups: untreated control and TMZ-treated cells at their respective IC50 concentrations (Supplementary Figure S2B). Real-time PCR analysis revealed that TMZ induced the differential expression of AMPK subunits. AMPKγ2 exhibited the most pronounced transcriptional upregulation, with a significant increase in both U87MG (Figure 2A) and LN229 cell lines (Supplementary Figure S2C) following TMZ exposure, suggesting a stress-responsive activation pattern. In contrast, AMPKγ1 showed a decrease in the TMZ-treated group. HEK293T cells, a non-tumor embryonic kidney cell line, were used as a control experiment (Supplementary Figure S2D), representing no significant change in AMPKγ1 and γ2 after treatment with TMZ.

To determine whether these transcriptional trends were represented at the protein level, Western blotting was performed. Based on these findings, subsequent experiments were performed for TMZ (358 μM) for 8 h. Consistent with mRNA data, AMPKγ2 protein levels were markedly elevated upon TMZ treatment, and AMPKγ1 levels decreased (Figure 2B). In addition to total AMPK subunits, we assessed AMPK activation status by measuring phosphorylated AMPK (pAMPK). Quantification showed that pAMPK levels increased approximately 2.5- to 3-fold in TMZ-treated cells compared to the control (Figure 2C), indicating the robust activation of AMPK signaling upon TMZ exposure. Overall, AMPKγ2 was reduced in clinical glioma samples, yet it became actively upregulated in response to TMZ. TMZ selectively enhances AMPKγ2 expression at both the mRNA and protein levels, suggesting a γ2-driven AMPK response during TMZ-induced cellular stress. These findings indicate that γ2 represents a more treatment-responsive AMPK isoform and may play a key role in shifting AMPK towards a resistant state during chemotherapy.

### 3.3. Overexpression of AMPKγ2 Reduces TMZ Sensitivity in Glioblastoma Cell Lines

**Figure 3:**
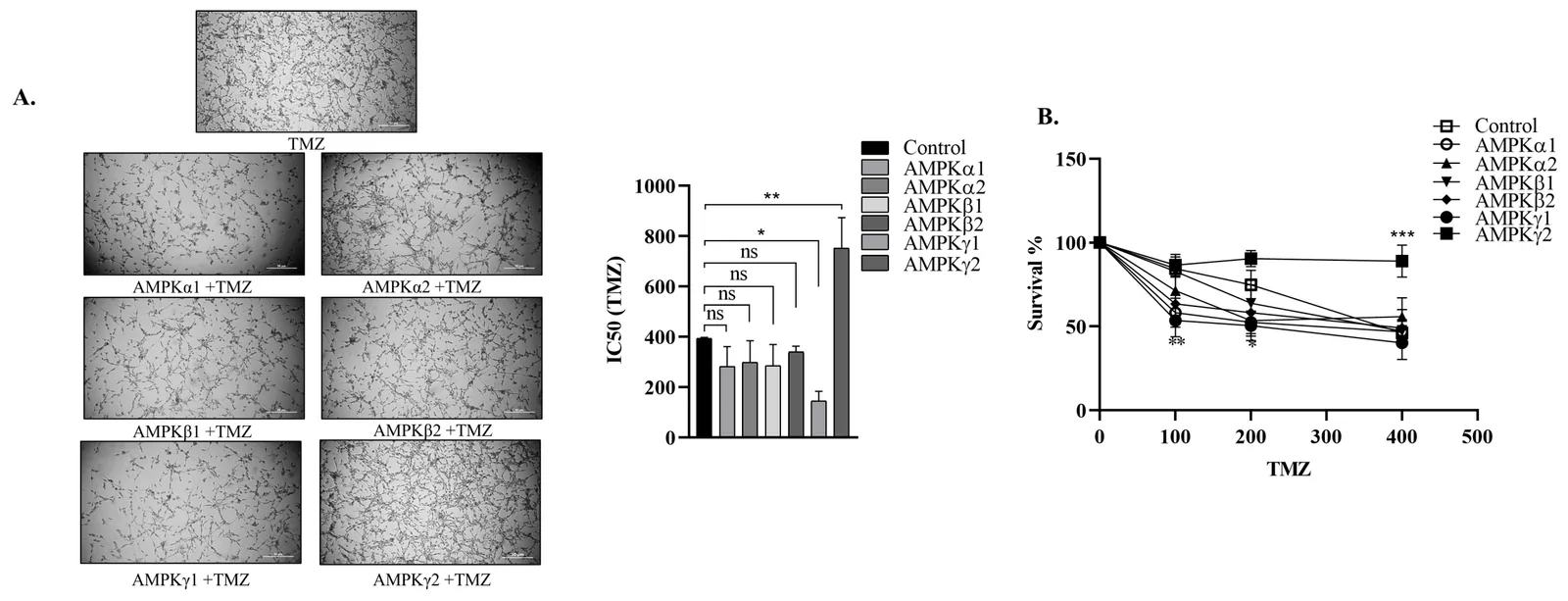
Overexpression of AMPKγ2 reduces TMZ sensitivity in GBM cell lines. (**A**) Phase-contrast images of U87MG cells treated with temozolomide (TMZ) following overexpression of AMPK subunits. Images illustrate morphological changes and cell density differences under control and TMZ-treated conditions. IC50 analysis of TMZ in U87MG cells following overexpression of individual AMPK subunits. Bar graph represents calculated IC50 values (μM), showing altered TMZ sensitivity upon modulation of AMPK subunits. (**B**) Dose-dependent curves showing effect of increasing TMZ concentrations on cell viability in U87MG cells expressing different AMPK subunits. Overexpression of AMPKγ2 results in increased TMZ resistance compared with other subunits. Data are presented as mean ± SD. Statistical significance was determined relative to control (* *p* < 0.05, ** *p* < 0.01; *** *p* < 0.001, ns = not significant).

We further investigated how individual AMPK subunits affect TMZ-induced cytotoxicity at increasing concentrations of the drug in glioblastoma cell lines. U87MG and LN229 cells were first transfected with AMPK subunit plasmids. After overexpressing each AMPK subunit, cells were treated with increasing doses of TMZ (100, 200, and 400 μM), and cell viability was evaluated using the MTT assay. Phase-contrast images (Figure 3A) revealed that cells overexpressing AMPKγ2 followed by TMZ treatment retained higher cell density and confluency with more visible adherent cells compared to TMZ-treated control and other subunit-transfected conditions. The concentration-dependent viability curves (Figure 3B) showed that the overexpression of AMPKα1, AMPKα2, AMPKβ1, AMPKβ2, and AMPKγ1 further intensified these effects, resulting in a pronounced loss of confluency even at lower TMZ concentrations and extensive cell death, consistent with the known role of AMPKα-β complexes in amplifying metabolic stress and promoting apoptosis under chemotherapeutic pressure. In contrast, the overexpression of AMPKγ2 showed a clear protective effect, preserving cell morphology and supporting significantly higher cell viability compared to TMZ alone. A similar protective trend was observed in LN229 cells (Supplementary Figure S3), where γ2-overexpressing cells exhibited minimal reduction in viability even at 400 μM TMZ, while other subunits showed substantial dose-dependent cell death. This indicates a γ2-specific survival advantage. These data suggest that while AMPKα and β subunits sensitize GBM cells to TMZ-induced stress, AMPKγ2 uniquely confers a cytoprotective response, supporting its potential role in modulating chemotherapy resistance.

### 3.4. TMZ Treatment in GBM Cells Exhibits A Distinct AMPK γ-Subunit Profile

**Figure 4:**
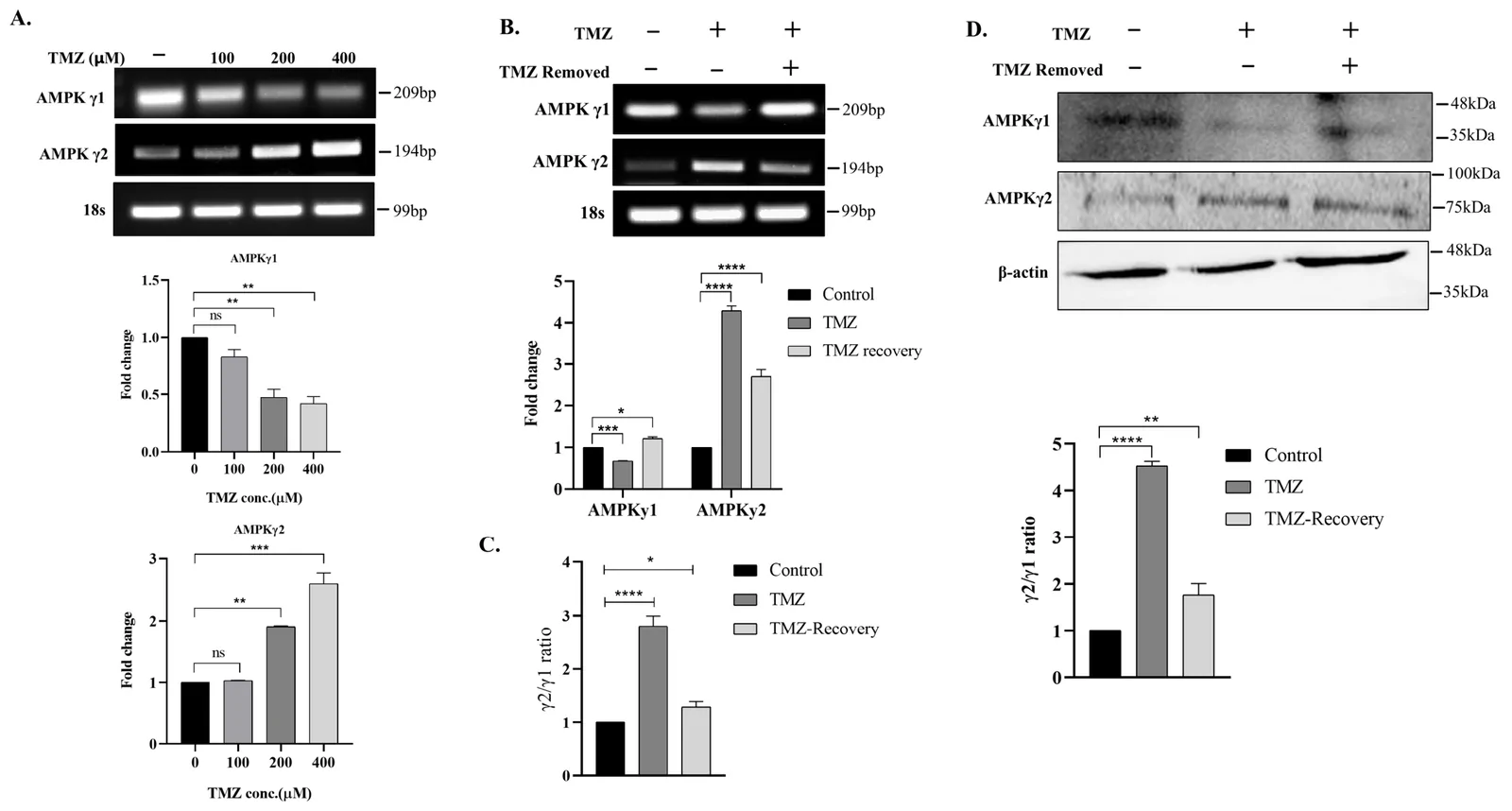
TMZ treatment in GBM cells exhibits a distinct AMPK γ-subunit profile. (**A**) Semi-quantitative RT-PCR analysis of AMPKγ1 and AMPKγ2 expression in U87MG cells treated with increasing concentrations of TMZ (0, 100, 200, and 400 μM). Representative gel images and densitometric quantification, normalized to 18s rRNA, are shown. (**B**) RT-PCR analysis of γ-subunit expression in U87MG cells under control, TMZ treatment, and TMZ recovery conditions (drug removal). Quantification demonstrates reversible modulation of γ-subunit expression following TMZ exposure. (**C**) Ratio of AMPKγ2/γ1 expression calculated from densitometric analysis, highlighting TMZ-induced shift in γ-subunit profile. (**D**) Western blot analysis of γ-subunit protein level under control, TMZ, and recovery conditions in U87MG cells. B-actin was used as control. Bar graph shows densitometric quantification of protein normalized to β-actin. Data are presented as mean ± SD of three biological repeats. Statistical significance was determined relative to control (* *p* < 0.05, ** *p* < 0.01, *** *p* < 0.001, **** *p* < 0.0001, ns = not significant).

To investigate the role of AMPK in response to TMZ treatment in GBM, U87MG cells were treated with increasing concentrations of TMZ, and the expression of the AMPK γ subunit was examined. Semi-quantitative and real-time PCR analysis revealed a dose-dependent alteration in γ-subunit expression (Figure 4A). While AMPKγ1 expression gradually decreased with increasing TMZ concentrations, AMPKγ2 expression showed a marked increase. Quantitative analysis confirmed a significant reduction in AMPKγ1 at higher TMZ doses (200–400 μM), whereas AMPKγ2 expression increased significantly compared to untreated controls. To further assess whether these changes were reversible, cells were analyzed following TMZ withdrawal (recovery condition). As shown in Figure 4B, TMZ treatment resulted in a significant decrease in AMPKγ1 transcript levels, whereas AMPKγ2 expression increased markedly. Upon the removal of TMZ, the partial restoration of γ1 expression and a reduction in γ2 levels were observed. Quantitative analysis demonstrated that TMZ treatment significantly suppressed AMPKγ1 while inducing AMPKγ2 expression compared with control cells. The γ2/γ1 expression ratio revealed a pronounced shift in AMPK regulatory subunit composition (Figure 4C). TMZ-treated cells exhibited nearly a three-fold increase in the γ2/γ1 ratio relative to untreated controls, indicating a substantial alteration in the AMPK subunit balance following chemotherapy exposure. These transcriptional changes were further validated at the protein level by Western blot analysis (Figure 4D). Consistent with the mRNA data, TMZ treatment reduced AMPKγ1 protein levels while significantly increasing AMPKγ2 expression. Upon drug removal, the partial normalization of γ1 and γ2 protein levels was observed. Densitometric quantification confirmed a significant decrease in γ1 and a corresponding increase in γ2 following TMZ treatment.

This transcriptional regulation was further validated using an independent transcriptomic dataset (GSE158772). The analysis of this dataset confirmed the differential expression of AMPK γ subunits following TMZ treatment. Consistent with our cellular models, *PRKAG2* showed a significant upregulation (Log2FC = 2.53), although *PRKAG1* was also elevated (Log2FC = 1.52) but not as robustly as *PRKAG2*. In contrast, *PRKAG3* (AMPKγ3) expression remained low and was not significantly altered (Supplementary Figure S4A). To identify the biological processes and pathways affected by TMZ treatment, we performed pathway enrichment analysis on the transcriptomic data. Among the top 20 significantly upregulated pathways (Supplementary Figure S4B), we observed a strong enrichment in metabolic and stress-responsive signaling cascades, including the AGE-RAGE signaling pathway in diabetic complications, diabetic cardiomyopathy, and energetics. Notably, multiple inflammatory and immune-related pathways were also prominently upregulated, such as the IL-17 signaling pathway, TNF signaling pathway, NF-kappa B signaling pathway, JAK-STAT signaling pathway, and NOD-like receptor signaling pathway. This suggests that TMZ-induced AMPK activation may be coupled with a pro-inflammatory transcriptional program. Additionally, pathways related to structural remodeling, including the cytoskeleton in muscle cells and ECM–receptor interaction, were among the most significantly upregulated. Conversely, the examination of the top 20 downregulated pathways (Supplementary Figure S4C) revealed the suppression of processes associated with specialized metabolic functions and tissue homeostasis, further supporting a metabolic shift in response to TMZ-induced stress. The significance and gene count for these enriched pathways are summarized in Supplementary Figure S4B,C.

### 3.5. Silencing AMPKγ2 Sensitizes Glioblastoma Cell Lines to TMZ

**Figure 5:**
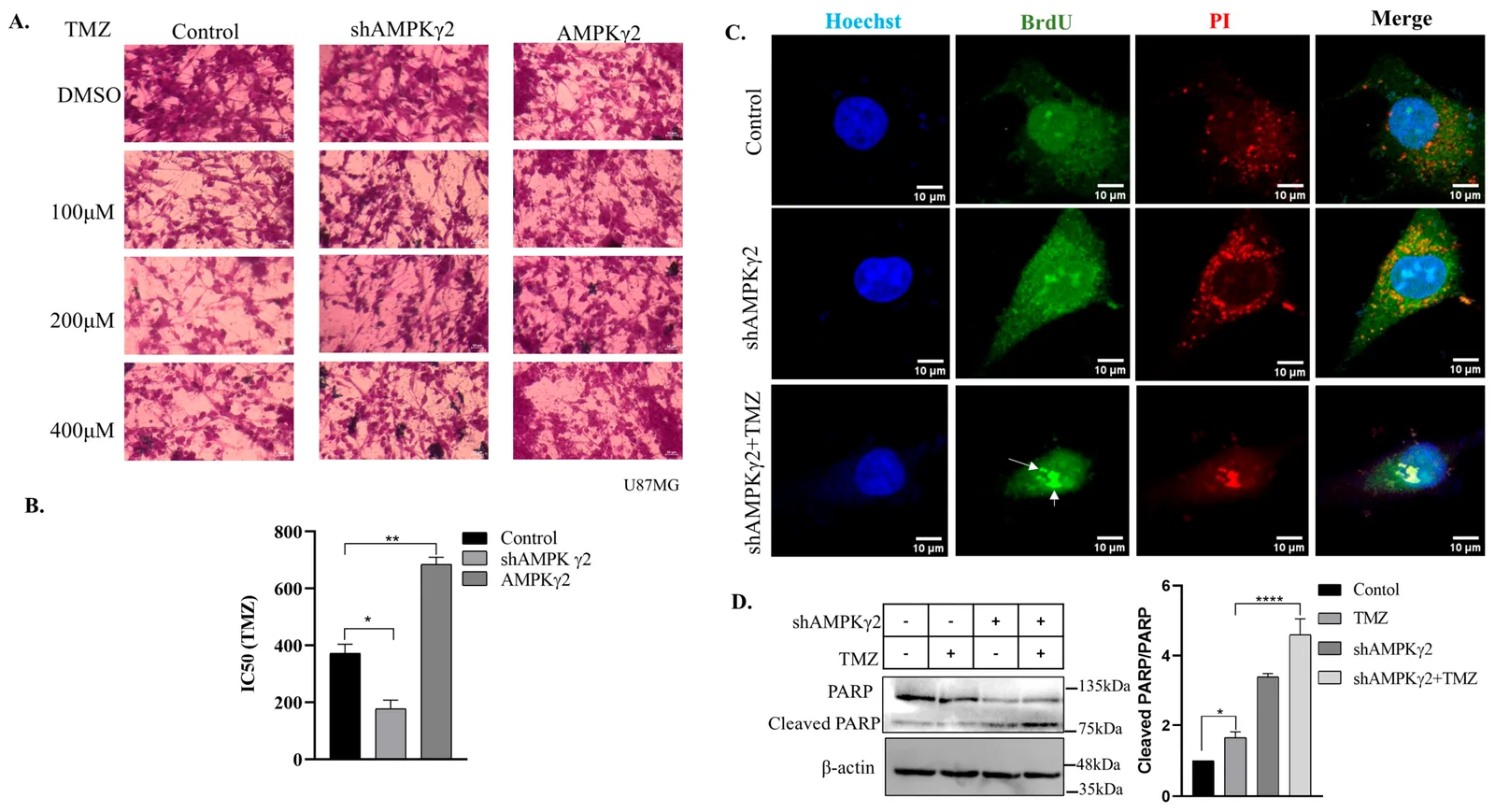
Silencing AMPKγ2 sensitizes GBM cells, while overexpression induces resistance to TMZ. (**A**) Representative crystal violet-stained images of U87MG cells transfected with shAMPKγ2 or AMPKγ2 overexpression following treatment with increasing concentrations of TMZ (0, 100, 200, and 400 μM). Silencing AMPKγ2 enhanced TMZ-induced growth inhibition, whereas AMPKγ2 overexpression promoted cell survival. Scale bar = 50 μm. (**B**) Quantification of TMZ IC_50_ values in control, shAMPKγ2-, and AMPKγ2-overexpressing U87MG cells. AMPKγ2 knockdown significantly reduced IC_50_ of TMZ, indicating increased chemosensitivity, while AMPKγ2 overexpression increased IC_50_, suggesting enhanced resistance to TMZ. Data are presented as mean ± SD. * *p* < 0.05, ** *p* < 0.01. (**C**) Representative confocal microscopy images of BrdU incorporation assay in control, shAMPKγ2-, and shAMPKγ2 + TMZ-treated U87MG cells. Nuclei were stained with Hoechst (blue); apoptotic cells were detected by BrdU (green) and propidium iodide (red) staining. Scale bar = 10 μm. (**D**) Representative Western blot showing PARP cleavage in control, TMZ-treated, shAMPKγ2-treated, and shAMPKγ2 + TMZ-treated U87MG cells, with β-actin used as loading control. Quantification of cleaved PARP/PARP ratio shows significant increase in PARP cleavage following combined AMPKγ2 knockdown and TMZ treatment, indicating enhanced apoptotic cell death. Data are presented as mean ± SD. * *p* < 0.05, **** *p* < 0.0001.

To validate the observation that AMPKγ2 overexpression reduces TMZ sensitivity, we next examined whether silencing AMPKγ2 expression levels could directly modulate the concentration-dependent cytotoxicity response to TMZ. Semi-quantitative PCR analysis confirmed the efficient modulation of AMPKγ2 expression. AMPKγ2 transcript levels were reduced in shAMPKγ2-transfected cells and elevated in AMPKγ2-overexpressing cells (Supplementary Figure S5A). Further validation across six experimental conditions (control, TMZ, γ2 knockdown, γ2 knockdown +TMZ, γ2 overexpression, γ2 overexpression + TMZ) showed distinct expression patterns (Supplementary Figure S5B), where AMPKγ2 expression increased upon TMZ treatment, decreased in knockdown cells, and was markedly elevated in overexpressing cells, with the highest expression observed in the TMZ-treated γ2-overexpressing cells. Quantitative analysis using MTT (Figure 5B, Supplementary Figure S5D) supported these trends, confirming the successful knockdown and overexpression of AMPKγ2. U87MG and LN229 cell lines were transfected to knock down AMPKγ2 using the shAMPKγ2 plasmid and overexpressed via the AMPKγ2 plasmid, followed by treatment with increasing TMZ concentrations (Figure 5A and Supplementary Figure S5C). As shown in crystal violet-stained phase-contrast images in control cells, TMZ induced a progressive reduction in cell density and increased cellular rounding in a dose-dependent manner. This cytotoxic effect was markedly amplified in knockdown cells, which showed extensive cell shrinkage and detachment even at 100–200 μM TMZ, indicating enhanced drug vulnerability. In contrast, AMPKγ2-overexpressed cells retained higher confluency and maintained a spindle-like morphology across all TMZ concentrations. Consistent with these phenotypic observations, cell viability measurements demonstrated that knockdown cells exhibited a decline in survival with increasing TMZ doses, whereas overexpressing cells maintained significant viability at 200–400 μM. To confirm that the observed reduction in cell viability was driven by apoptosis, we performed a BrdU TUNEL assay in U87MG cells (Figure 5C). Following treatment with shAMPKγ2 and TMZ, we observed an intense labeling in the nucleus, which reflects extensive DNA strand breaks from shAMPKγ2-treated cells in combination with TMZ-treated U87MG cells. This distinct green signal from the nucleus confirms that the combined treatment indices induce DNA damage-dependent apoptosis in the U87MG cells. The co-localization of the PI red signal with the BrdU green signal confirmed widespread apoptotic chromatin condensation and nuclear degradation. Further, to investigate apoptosis-associated changes, we examined the expression of PARP, a key DNA damage repair protein. Western blot analysis showed increased cleaved PARP and reduced total PARP expression in AMPKγ2-silenced cells following TMZ treatment (Figure 5D).

### 3.6. AMPKγ2 Knockdown Diminishes While Overexpression Enhances AMPK Activation Under TMZ Treatment

**Figure 6:**
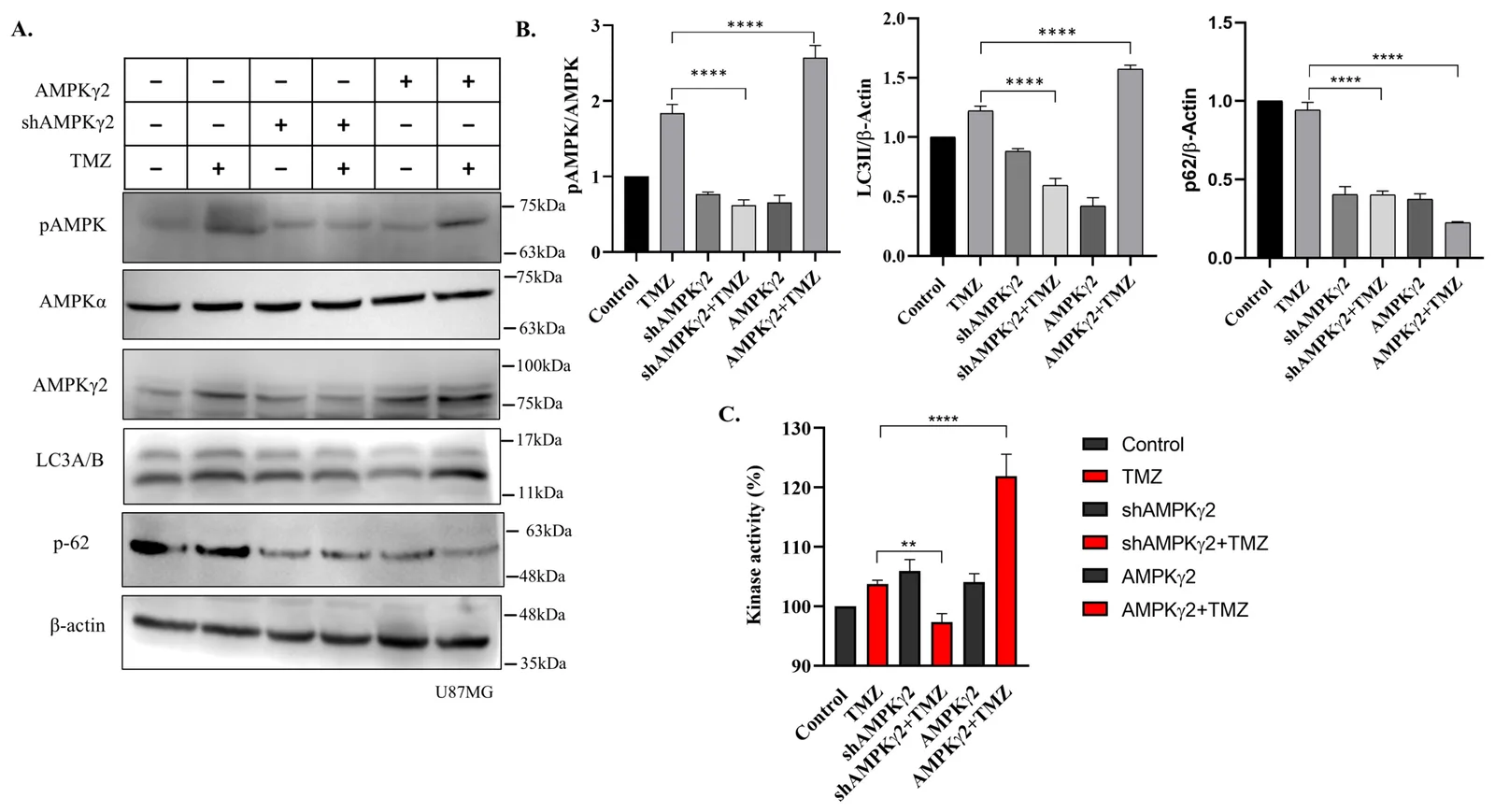
AMPKγ2 knockdown diminishes while overexpression enhances AMPK activation under TMZ treatment. (**A**) Western blot analysis showing the expression of pAMPK, total AMPKα, AMPKγ2, LC3A/B, p-62, and β-actin in U87MG cells under six experimental conditions: control, TMZ treatment, AMPKγ2 knockdown (shAMPKγ2), and AMPKγ2 overexpression (AMPKγ2), alone or in combination with TMZ. The treatment matrix indicating the presence (+) or absence (−) of AMPKγ2, shAMPKγ2, and TMZ is shown above the blots. β-actin serves as the loading control. (**B**) The densitometric quantification of protein levels normalized to β-actin. The pAMPK/AMPK ratio indicating AMPK activation status. The LC3II/β-actin and p-62/β-actin ratio as a marker of autophagy. Statistical significance was determined using an ANOVA. (**C**) AMPK activity assay showing percentage kinase activity under control and TMZ-treated conditions in shAMPKγ2- and AMPKγ2-overexpressing cells. Data are presented as the mean ± SD of three biological repeats. ** *p* < 0.01, **** *p* < 0.0001).

To investigate whether AMPKγ2 modulated AMPK activation under stress, U87MG cells were subjected to γ2 knockdown and overexpression, followed by TMZ treatment. Western blot analysis (Figure 6A) showed that pAMPK levels increased after TMZ exposure in control cells, consistent with initial time course observations. Notably, γ2 knockdown attenuated this TMZ-induced rise in pAMPK, whereas γ2 overexpression markedly enhanced AMPK phosphorylation, indicating that γ2 expression directly influences AMPK activation efficiently. Total AMPK levels remained stable across all groups, confirming that changes in phosphorylation were not due to altered AMPK abundance. AMPKγ2 protein levels corresponded appropriately to knockdown and overexpression conditions. We further evaluated autophagy by monitoring LC3A/B. LC3 (microtubule-associated protein 1 light chain 3) is a key autophagy marker, and its conversion from LC3-I to LC3-II reflects autophagosome formation, serving as an indicator of autophagic activity [40]. This conversion occurs via lipidation, wherein LC3-I is conjugated to phosphatidylethanolamine to form the membrane-bound LC3-II [41]. TMZ treatment increased the lipidated LC3-II form, and this shift was more prominent under γ2 overexpression, whereas γ2 knockdown blunted the LC3 conversion pattern, implying an AMPKγ2-dependent enhancement in autophagy signaling. We next examined p62/SQSTM1, a selective autophagy receptor that binds ubiquitinated cargo and delivers it to autophagosomes for lysosomal degradation [42]. Since p62 is itself degraded during autophagy, reduced p62 protein levels are commonly associated with autophagic activity [43]. TMZ treatment altered p62 expression prominently in AMPKγ2-overexpressing cells, suggesting the modulation of the autophagy pathway. The quantification of pAMPK normalized to AMPK showed the highest activation in γ2-overexpressing TMZ-treated cells (Figure 6B), supporting the Western blot observations. Finally, kinase activity assay demonstrated a similar trend (Figure 6C): γ2 knockdown reduced AMPK activity in response to TMZ, whereas γ2 overexpression significantly elevated kinase activity, particularly under TMZ treatment. To evaluate mitochondrial health, JC-1 staining was performed (Supplementary Figure S6). Control cells exhibit strong red fluorescence, indicating intact mitochondrial membrane potential. TMZ treatment led to a marked reduction in red fluorescence with a corresponding increase in the green signal, suggesting mitochondrial depolarization. In contrast, γ2 overexpression in TMZ-treated cells restored red fluorescence intensity, indicating improved mitochondrial membrane potential compared to TMZ alone. The quantitative analysis of the red/green fluorescence ratio further supported these observations, showing a significant decrease in TMZ-treated cells relative to control, while γ2 overexpression significantly increased the ratio under TMZ conditions. Together, these results demonstrate that AMPKγ2 amplifies TMZ-induced AMPK activation, positioning γ2 as the preferred and functionally dominant AMPK subunit during chemotherapy stress.

### 3.7. Co-Immunoprecipitation Confirms the Structural Remodeling of the AMPK Heterotrimer

**Figure 7:**
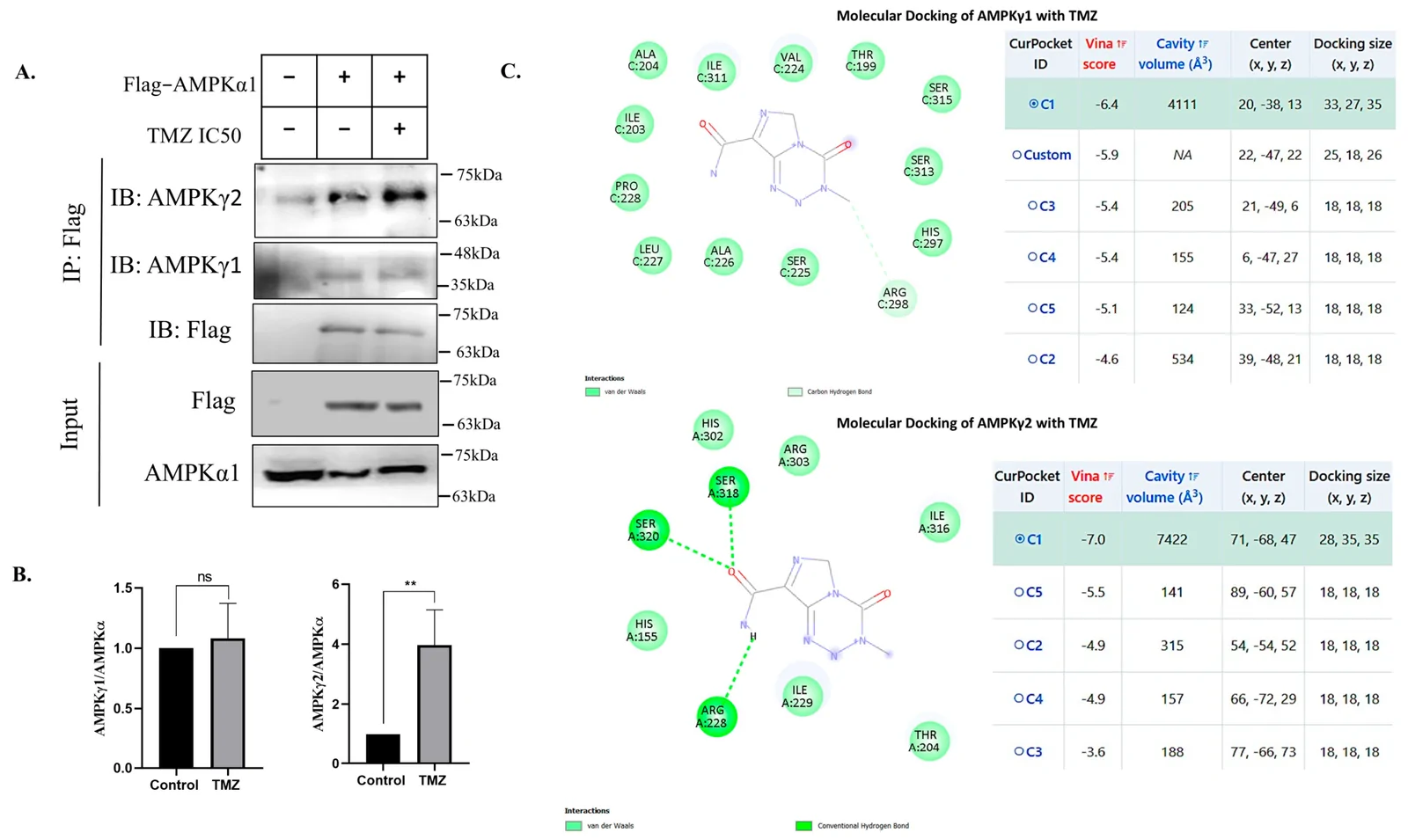
Co-immunoprecipitation confirms structural remodeling of AMPK heterotrimer. (**A**) Immunoblot of co-IP performed in U87MG cells under control and TMZ-treated conditions. AMPKα was immunoprecipitated, and association of regulatory subunits AMPKγ1 and AMPKγ2 within complex was analyzed by immunoblotting. Input lysates are shown to confirm equal protein expression across conditions. (**B**) γ2 and γ1 normalized using AMPKα input serve as indicator of subunit preference. Upon TMZ treatment, AMPKγ2 increases significantly, indicating shift in binding occupancy within AMPK heterotrimer. ** *p* < 0.01, ns = not significant. (**C**) Molecular docking analysis of TMZ with AMPKγ isoforms. Predicted binding poses of TMZ within AMPKγ2 structure reveal stable interaction within hydrophobic pocket involving residues ALA204, ILE224, VAL226, THR227, LEU228, ALA229, PRO311, and SER315, along with carbon–hydrogen bond with SER225. Comparative docking shows stronger binding affinity of TMZ to AMPKγ2 (ΔG = −7.0 kcal/mol) relative to AMPKγ1 (ΔG = −6.4 kcal/mol). In the 2D interaction diagrams, light green residues represent van-der Waals interactions, whereas green dashed lines indicate conventional hydrogen-bond interactions.

We next asked whether the observed expression changes resulted in the formation of new AMPK complexes. To establish whether AMPKγ2 serves as the dominant regulatory subunit engaged during TMZ-induced AMPK activation, we performed co-immunoprecipitation (co-IP) assays in U87MG cells treated with TMZ (Figure 7A). AMPKα was immunoprecipitated from control and TMZ-treated lysates, and the presence of AMPKγ1 and AMPKγ2 in the AMPKα complex was examined to assess subunit-specific association. In untreated cells, both γ1 and γ2 were detectable in the AMPKα immunoprecipitated, although γ1 exhibited slightly higher basal association. In parental cells, the AMPK α1 catalytic subunit was primarily associated with the γ1 isoform. However, in TMZ-resistant cells, there was a robust shift in binding occupancy: α1’s association with γ1 significantly decreased, while its association with γ2 increased by five times (Figure 7B). These data provide definitive evidence that the AMPK heterotrimer undergoes physical remodeling (a “subunit switch”) during the acquisition of TMZ resistance. Following TMZ treatment, a clear shift in subunit preference emerged. AMPKγ2 co-precipitation increased markedly, indicating the enhanced recruitment of γ2 to the AMPKα complex under metabolic stress. In contrast, γ1 association showed minimal change, suggesting that γ1 does not respond dynamically to TMZ-induced activation cues. The input controls confirmed in co-IP intensities reflected altered binding rather than differences in protein abundance. Importantly, the increase in γ2 association paralleled the heightened AMPK phosphorylation observed under TMZ treatment, supporting a model in which γ2 facilitates or stabilizes stress-induced AMPK activation. The preferential interaction between AMPKγ2 and AMPKα reinforces earlier findings from overexpression and knockdown experiments, where γ2 abundance strongly correlated with enhanced pAMPK levels and kinase activity. These co-IP results provide biochemical evidence that AMPKγ2 is selectively engaged during TMZ-triggered AMPK activation, thereby functioning as the preferred regulatory subunit orchestrating AMPK signaling under chemotherapeutic stress.

To complement these co-IP observations, we next performed molecular docking simulations to assess whether TMZ directly interacts with AMPKγ isoforms and whether structural determinants could account for the preferential engagement of AMPKγ2 in TMZ-treated cells (Figure 7C). Using a curated structural model of the AMPK γ-subunit, TMZ was blindly docked into the predicted binding pockets of both the γ1 and γ2 isoforms. Docking scores and interaction profiles were compared to evaluate isoform-specific binding preferences. In silico analysis revealed that TMZ binds to AMPKγ2 with a more favorable binding energy (ΔG = −7.0 kcal/mol) compared to AMPKγ1 (ΔG = −6.4 kcal/mol), suggesting enhanced affinity for the γ2 isoform. The closer inspection of the docking pose for AMPKγ2 revealed that TMZ is stabilized within a hydrophobic cleft through multiple non-covalent interactions. The key residues involved included van der Waals contacts with ALA:204, ILE:224, VAL:226, THR:227, LEU:228, ALA:229, PRO:311, and SER:315. Additionally, a carbon–hydrogen bond was observed with SER:225, further stabilizing the ligand within the binding pocket. These interactions collectively support a model in which TMZ binds directly to AMPKγ2, potentially modulating its conformation or stabilizing its association with the AMPKα catalytic subunit. In contrast, the docking of TMZ to AMPKγ1 yielded fewer favorable contacts and a less complementary binding interface, consistent with its lower binding affinity and the reduced functional engagement observed in co-IP experiments. Together, these in silico findings provide a structural rationale for the TMZ-induced subunit switch observed in Figure 7A. The preferential docking of TMZ to AMPKγ2 may facilitate the recruitment and stabilization of this regulatory subunit within the AMPK heterotrimer, thereby promoting sustained kinase activation under conditions of chemotherapeutic stress.

To further explore the structural basis underlying isoform-specific interactions within the AMPK heterotrimer, we performed molecular docking simulations to compare the binding of ATP to the AMPKγ1 and AMPKγ2 subunits (Supplementary Figure S7). Given that AMPK functions as a cellular energy sensor primarily regulated by adenine nucleotide binding to its γ subunit, we reasoned that differential ATP affinity between γ isoforms could contribute to the functional divergence observed in TMZ-resistant cells. Docking analyses revealed marked differences in ATP binding parameters between the two isoforms. The AMPKγ1 subunit exhibited a Vina score of −9.1 kcal/mol, indicating robust ATP binding within a predicted cavity. In contrast, ATP docking to AMPKγ2 yielded a less favorable binding energy of −7.6 kcal/mol, despite a substantially larger cavity volume. These findings suggest that while AMPKγ2 possesses a more expansive potential binding pocket, its structural configuration confers reduced ATP affinity relative to AMPKγ1 under basal conditions. The differential ATP binding affinities observed here align with the established role of AMPKγ1 as a high-affinity ATP-binding module under energy-replete conditions. Conversely, the lower ATP affinity of AMPKγ2 may render this isoform more responsive to fluctuations in cellular energy status, particularly under conditions of metabolic stress such as TMZ exposure. This structural distinction provides a plausible mechanism for the preferential engagement of AMPKγ2 during TMZ-induced AMPK activation, as the reduced ATP occupancy in γ2 may facilitate allosteric activation by AMP or ADP when ATP levels decline.

To gain insight into the transcriptional landscape associated with AMPK γ-subunit expression in glioblastoma, we analyzed publicly available tumor datasets to identify genes whose expression correlates with *PRKAG1* and *PRKAG2*, the genes encoding the AMPKγ1 and AMPKγ2 regulatory subunits, respectively (Supplementary Figures S8 and S9). This approach was undertaken to infer potential coregulated pathways and biological processes linked to each isoform. An analysis of *PRKAG1*-correlated genes in glioblastoma specimens revealed a distinct set of positively associated transcripts enriched for components of cellular housekeeping and metabolic complexes (Supplementary Figure S8). Among the top positive correlates were VPS29 (ρ = 0.8), SNAPIN (ρ = 0.8), and LAMTOR5 (ρ = 0.77), all of which participate in endosomal trafficking and mTOR signaling. Additional positively correlated genes included multiple subunits of the oxidative phosphorylation machinery (NDUFA4, NDUFC2, NDUFA12), components of the COP9 signalosome (COPS4), and assembly factors for mitochondrial complexes (COX14, NFU1). This profile suggests that PRKAG1 expression aligns with genes governing fundamental metabolic infrastructure, consistent with the canonical role of AMPKγ1 in energy homeostasis under basal conditions. Conversely, genes negatively correlated with *PRKAG1* included transcriptional regulators such as SRRM2 (ρ = −0.71), ZNF646 (ρ = −0.71), and PRRC2B (ρ = −0.69), as well as chromatin modifiers including KDM6B (ρ = −0.66) and KMT2B (ρ = −0.65), indicating an inverse relationship between *PRKAG1* expression and certain transcriptional regulatory networks.

In contrast, *PRKAG2*-correlated genes exhibited a markedly different expression signature (Supplementary Figure S9). The top positive correlates included ETNPPL (ρ = 0.58), SLC14A1 (ρ = 0.57), and PPP1R1B (ρ = 0.55), genes involved in metabolic adaptation and stress response. Several additional positively associated transcripts such as GCLC (ρ = 0.52), a rate-limiting enzyme in glutathione synthesis, and PEBP4 (ρ = 0.51), implicated in cell survival signaling, point toward a coordinated program linked to oxidative stress management and therapeutic resistance. Notably, genes negatively correlated with *PRKAG2* included those encoding endoplasmic reticulum chaperones (PDIA3, PDIA6, HSP90B1) and components of the extracellular matrix (LAMC1, COL9A3), as well as immune-modulatory molecules such as CD276 (ρ = −0.47). This inverse correlation suggests that high *PRKAG2* expression may be associated with the repression of ER stress and matrix remodeling pathways. Collectively, these correlation analyses reveal that *PRKAG1* and *PRKAG2* are embedded within distinct transcriptional networks in glioblastoma. While *PRKAG1* correlates with genes maintaining core metabolic and proteostatic functions, *PRKAG2* expression aligns with a stress-adaptive transcriptional program. This divergence provides a transcriptomic rationale for the functional switch toward γ2-dominant AMPK signaling observed under TMZ-induced metabolic stress.

To explore whether AMPKγ may influence upstream AMPKs, correlation analyses were performed using glioma datasets. Correlation analysis revealed a positive association between PRKAG1 (Supplementary Figure S10A) and the upstream AMPK kinases STK11 (R = 0.64) and CAMKK2 (R = 0.42), suggesting that AMPK subunit expression may be coordinated with upstream kinase pathways. However, PRKAG2 expression showed a weak positive correlation with SKT11 (LKB1) (R = 0.39), whereas virtually no correlation was observed with CAMKK2 (R = 0.091) (Supplementary Figure S10B).

## 4. Discussion

### 4.1. Clinical Implications: Beyond General AMPK Activation

Historically, researchers have attempted to treat cancer using global AMPK activators (such as metformin) [44]. However, our data suggests that a more nuanced approach is required. If GBM cells survive by switching to a γ2-specific complex, a general activator might inadvertently help resistant cells [45]. Instead, our study points toward isoform-specific inhibition or the prevention of the “switch” itself as a superior therapeutic strategy to resensitize GBM to TMZ. Glioblastoma (GBM) is a highly aggressive brain tumor with rapid progression and strong resistance to conventional chemotherapy [46]. AMP-activated protein kinase (AMPK) is a heterotrimeric αβγ complex that regulates cellular energy balance, with isoform diversity contributing to its activity and localization [47]. In this study, we analyzed the expression of AMPK subunits in GBM cell lines and evaluated their response to the chemotherapeutic drug TMZ. Using publicly available transcriptomic datasets and validation in GBM and LGG patient samples, we observed a fundamental variation in the expression of the γ1 and γ2 subunits. The in silico analysis of γ2 subunit expression was validated using q-PCR, which showed a marked downregulation in GBM compared to the NB. Previous studies have shown differences in AMPK subunit expression affecting AMPK activation [48,49,50]. We observed that although GBM samples showed a lower expression level of AMPKγ2, treatment with TMZ led to a significantly increased expression level of AMPKγ2, accompanied with elevated p-AMPK levels, indicating enhanced pathway activation under chemotherapy stress. Previous studies have already confirmed that TMZ induces AMPK activation in the primary cultured GBM cell lines [39].

To further evaluate the importance of AMPK subunits in modulating TMZ response, U87MG and LN229 cells were transfected with different AMPK subunits. Transfection with AMPKα and AMPKβ and treatment with different concentrations of TMZ led to increased sensitivity to TMZ, but cells transfected with AMPKγ2 showed decreased sensitivity to TMZ. Further, to confirm switching between the γ1 and γ2 subunits, we treated GBM cells with TMZ for 24 h and then replaced the culture media with fresh media to analyze expression patterns during recovery. A significant increase and decrease in the γ2 and γ1 subunits respectively upon TMZ treatment were reversed during the removal of TMZ, thus demonstrating a distinct AMPKγ expression profile during TMZ treatment. A significantly low expression level in the AMPKγ2 subunit in the recovery period justified the switching of the γ2 to γ1 subunit under recovery. These findings were also observed with the GEO dataset (GSE158772), although γ1 and γ2 both showed an increase in expression upon TMZ treatment, but γ2 showed a 3-fold-higher increase, demonstrating its cytoprotective role during chemotherapy. Corroborating this, there are previous studies describing the cytoprotective role of the γ2 subunit in myocardial Ischemia/reperfusion injury and breast cancer [51,52]. To confirm the cytoprotective role of the γ2 subunit during TMZ treatment, we silenced and overexpressed AMPKγ2 in GBM cell lines. We observed a significantly reduced and enhanced survival of TMZ-treated GBM cells upon AMPKγ2 silencing and overexpression respectively. Furthermore, a significant change in TMZ IC50 was observed, suggesting that silencing the AMPKγ2 subunit sensitized GBM cells, whereas overexpression made them resistant to TMZ, leading to increased cell viability.

Moreover, TMZ treatment in AMPKγ2-overexpressing cells caused a significant increase in pAMPK levels as compared to TMZ treatment alone. On the contrary, the knockdown of AMPKγ2 abolished TMZ-induced AMPK activation, highlighting the crucial role of AMPKγ2 in mediating AMPK activation under chemotherapeutic stress. A previous study reported that the depletion of γ1, but not γ2, drastically reduced the metformin activation of AMPK [53]. Thus, the γ1 and γ2 subunits might play a context-dependent role in fine-tuning AMPK activation under different conditions. The unique role of AMPKγ2 was further validated by the co-IP experiment confirming the preferential binding of the γ2 subunit with the AMPKαβ complex under TMZ treatment. Thus, the isoform-specific function of AMPKγ subunits plays an important role in AMPK activation. These findings suggest that AMPKγ2 might play a key role in GBM chemoresistance, and the targeted modulation of AMPKγ2 may provide a promising strategy to sensitize tumor cells to TMZ and improve therapeutic outcomes.

### 4.2. The “Energy Sensor” Sensitivity Hypothesis

The most significant finding of this study is that the AMPK heterotrimer is not a static entity but a dynamic sensor that adapts to chemotherapy. The γ subunits contain four cystathionine beta-synthase (CBS) domains, known as Bateman domains, which serve as the regulatory heart of the complex by binding AMP, ADP, or ATP [54]. Our results suggest that the transition from γ1 to γ2 in glioblastoma (GBM) cells essentially recalibrates the cellular fuel gauge. The literature indicates that γ2-containing complexes often exhibit different affinities for AMP and are less sensitive to inhibition by ATP compared to γ1 [48]. In the context of TMZ treatment, which induces DNA damage and subsequent ATP depletion, this switch likely allows AMPK to remain active (phosphorylated) even when the energy crisis is severe, ensuring that survival-promoting pathways like fatty acid oxidation and autophagy are maintained.

### 4.3. Bypassing the “Death Threshold”

In parental γ1-dominant cells, the rapid drop in ATP following TMZ exposure leads to the failure of AMPK to sustain metabolic homeostasis, resulting in metabolic collapse and apoptosis. By remodeling the complex to include the γ2 isoform, resistant cells effectively lower their “death threshold.” This isoform diversity provides a selective advantage, allowing the tumor to navigate the energetic stress of alkylating agents that would otherwise be lethal (Table 3).

## 5. Conclusions

In summary, our findings demonstrate that glioblastoma cells adapt to TMZ-induced stress through the dynamic remodeling of the AMPK complex, characterized by a switch from the γ1 to the γ2 subunit. This transition promotes sustained AMPK activation, enhances metabolic adaptation, and increases resistance to TMZ, whereas silencing AMPKγ2 sensitizes tumor cells to chemotherapy. Mechanistically, γ2-containing complexes appear to recalibrate cellular energy sensing, enabling GBM cells to survive under severe energetic stress. These results identify AMPKγ2 as a critical mediator of chemoresistance and highlight the isoform-specific targeting of AMPK, particularly the inhibition of AMPKγ2 or prevention of the γ1 to γ2 switch, as a promising therapeutic strategy for improving TMZ efficacy in glioblastoma.

## Figures and Tables

**Table 1:** List of GBM and LGG tissue samples.

S. No	Sample Code	Code Number	Gender	Age	Diagnosis
1	AIIMS-D/GBM/F/28	28	F	58	GBM
2	AIIMS-D/GBM/46	46	F	51	GBM
3	AIIMS-D/GBM/M/78	78	M	52	GBM
4	AIIMS-D/GBM/M/95	95	M	59	GBM
5	AIIMS-D/GBM/56	56	M	58	GBM
6	AIIMS-D/LGG/M/6	6	M	27	LGG
7	AIIMS-D/LGG/M/7	7	M	26	LGG
8	AIIMS-D/LGG/M/8	8	M	35	LGG
9	AIIMS-D/LGG/M/11	11	M	20	LGG
10	AIIMS-D/LGG/M/12	12	M	26	LGG

**Table 2:** List of primer sequences.

Gene ID	Forward Primer Seq (5′–3′)	Reverse Seq (3′–5′)	Annealing Temp. (°C)
*PRKAA1*	TTGAAACCTGAAAATGTCCTGCT	GGTGAGCCACAACTTGTTCTT	58
*PRKAA2*	CTGCTGGCTTACACAGACCA	GGCGAGGTGAAACTGAAGAC	59
*PRKAB1*	CCCTTGCTCAGGGTCCCTTT	CCCTCCGGGGCGTCTTAT	60
*PRKAB2*	CCACTGTTATCCGCTGGTCT	GAACTTGTATTGGTGCTCTC	60
*PRKAG1*	CAAGAGACCCCAGAATCCAA	CCTGCAGGGACGTATCAAAT	58
*PRKAG2*	CGTACCACAACATTGCCTTC	CTGGGTCACCGTGATATCTAG	58
*18S*	GAATCGAACCCTGATTCCCCGTC	CGGCGACGACCCATTCGAAC	99

**Table 3:** The biochemical basis of the γ2 subunit switch.

Feature	γ1-AMPK (Sensitive)	γ2-AMPK (Resistant)
ATP Sensitivity	High (inhibited easily by ATP)	Low (maintains activity during stress)
Metabolic State	Dependent on Glucose	High Mitochondrial Plasticity
Response to TMZ	Energy Collapse/Apoptosis	Metabolic Adaptation/Survival
Clinical Outcome	Chemosensitivity	Chemoresistance

## Data Availability

The raw data supporting the findings of this study are available from the corresponding author upon reasonable request.

## Supplementary Materials

The following supporting information can be downloaded at https://www.mdpi.com/article/10.3390/cells15171594/s1, Figure S1: (A) shows the correlation between AMPK subunits. (B,C) expression of *PRKAG2* in normal and tumor (GBM), and based on the patient’s age. (D,E) promoter methylation level in *PRKAG2* in normal and GBM patient samples, and based on the GBM, based on the patient’s age. (F) Protein expression of PRKAG2 in GBM; (G) shows methylation of PRKAG1 and PRKAG2 in normal and tumor tissues. Figure S2: (A) The IC50 of TMZ (μM) in U87MG (358 μM), blue line and LN229 (375 μM) red line, is represented in a line graph, *y*-axis represents cell survivability. (B) Represents AMPKγ1 and AMPKγ2 expression in control and TMZ-treated LN229 cells. (C) shows pAMPK vs. AMPK treated with TMZ at different times in U87MG to check AMPK activation. (D) qPCR of HEK293T cell showing no change in AMPKγ expression while treated with TMZ. Figure S3: (A) Representing phase contrast images of LN229 cells treated with TMZ along with overexpression of AMPK subunits. Figure S4: (A) The differential expression of AMPKγ subunits obtained from the transcriptomic dataset (GSE158772). (B,C) Represents pathway enrichment analysis of transcriptomic data. It represents the top 20 pathways that are significantly up- or downregulated. The size of the dot represents the number of genes, and the color represents −log10(FDR). Figure S5: (A) RT-PCR validation of AMPKγ2 knockdown (shAMPKγ2) and AMPKγ2 overexpression in U87MG cells. Expression levels were normalized to 18s rRNA. (B) Quantification of AMPKγ2 expression under control, TMZ treatment, AMPKγ2 knockdown and, overexpression, AMPKγ2 knockdown and overexpression with TMZ treatment conditions, demonstrating efficient modulation of γ2 expression in U87MG cells. (C) Representative crystal violet staining images of LN229 cells under control, AMPKγ2 knockdown (shAMPKγ2), and AMPKγ2 overexpression conditions following treatment with increasing concentrations of TMZ. (B) MTT quantification of TMZ IC50 values in LN229 cells across the indicated conditions. AMPKγ2 overexpression significantly increases the IC50, whereas AMPKγ2 knockdown lowers it. Data are presented as mean ± SEM, and statistical significance is indicated (* *p* < 0.05). Figure S6: JC-1 staining images showing mitochondrial membrane potential in U87MG cells under control, TMZ-treated, and AMPKγ2 overexpression with TMZ conditions. Red fluorescence indicates polarized (healthy) mitochondria, whereas green fluorescence indicates depolarized mitochondria. TMZ treatment markedly increases green and reduces red fluorescence, indicating mitochondrial depolarization. In contrast, AMPKγ2 overexpression partially restores mitochondrial membrane potential. Figure S7: Molecular docking of ATP with AMPKγ1 and γ2 subunits showing binding affinities, cavity volumes, and docking parameters. The docking result for the γ1 subunit indicates the highest binding affinity at cavity C1, with a Vina score of −9.1 kcal/mol. The docking result for the γ2 subunit shows −7.6 kcal/mol but a larger cavity volume. Figure S8: Top 20 positively and negatively correlated genes to *PRKAG1* (AMPKγ1) expression in glioblastoma. Spearman correlation analysis was performed to identify genes associated with *PRKAG1* expression. Positively correlated genes are shown in red bars and negatively correlated genes in blue bars. Correlation coefficients are shown for each gene, with positive correlations ranging from 0.74 to 0.80 and negative correlations ranging from −0.64 to −0.71. Figure S9: Top 20 positively and negatively correlated genes to *PRKAG2* (AMPKγ2) expression in glioblastoma. Spearman correlation analysis was performed to identify genes associated with *PRKAG1* expression. Positively correlated genes are shown in red bars and negatively correlated genes in blue bars. Correlation coefficients are shown for each gene, with positive correlations ranging from 0.5 to 0.58 and negative correlations ranging from −0.43 to −0.53. Figure S10: (A) Correlation analysis of *PRKAG1* vs. *SKT11* (R = 0.64) and *CAMKK2* (R = 0.42) shows positive correlation whereas with *PRAKG2* (B) *SKT1* (R = 0.39) showing weak positive correlation and with no correlation with *CAMKK2* (0.091).

## Abbreviations

GBM	Glioblastoma
LGG	Low-Grade Glioma
TMZ	Temozolomide
AMPK	AMP-Activated Protein Kinase
TCGA	The Cancer Genome Atlas
HPA	Human Protein Atlas
DMSO	Dimethyl Sulfoxide
FBS	Fetal Bovine Serum
DMEM	Dulbecco’s Modified Eagle Medium
GEO	Gene Expression Omnibus
