# Supplementary material for: AMPK γ-Subunit Isoform Switching Governs Temozolomide Resistance and Survival in Glioblastoma
Source: Cells. 2026 Sep 2;15(17):1594. doi: 10.3390/cells15171594 (PMC13564821; doi:10.3390/cells15171594)
Supplement: Supplementary file 1 [file cells-15-01594-s001.zip › cells-4363296-supplementary.pdf]

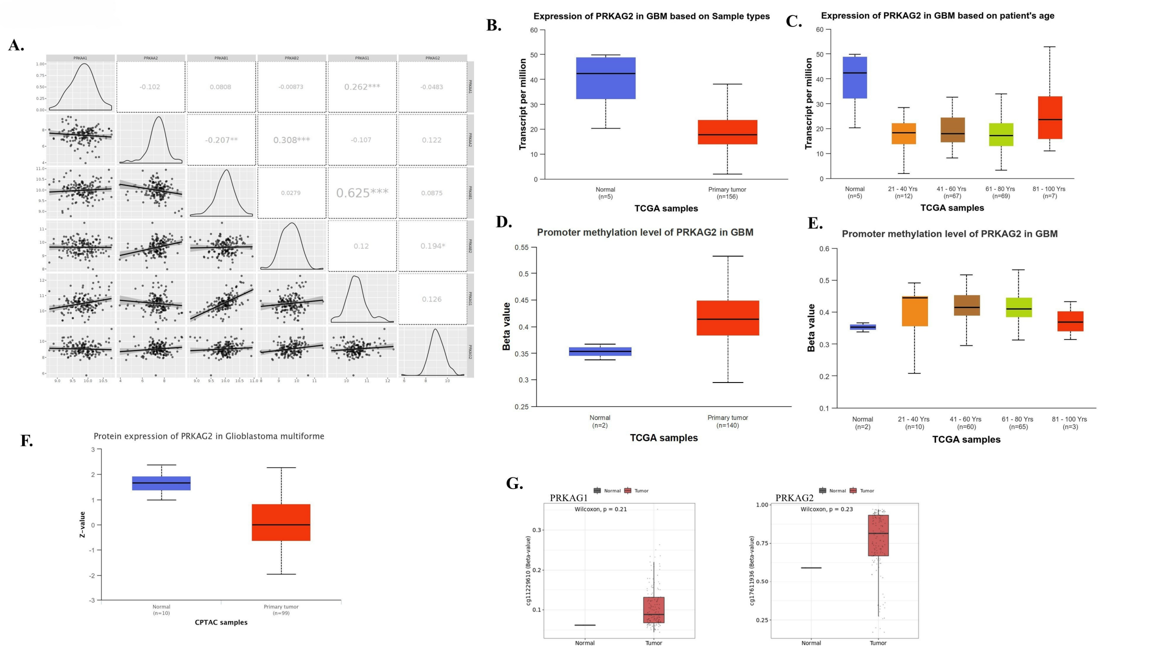

Figure S1: (A) shows the correlation between AMPK subunits. (B-C) expression of *PRKAG2* in normal and tumor (GBM), and based on the patient's age. (D-E) promoter methylation level in *PRKAG2* in normal and GBM patient samples, and based on the GBM, based on the patient's age. (F) Protein expression of PRKAG2 in GBM; (G) shows methylation of PRKAG1 and PRKAG2 in normal and tumor tissues.

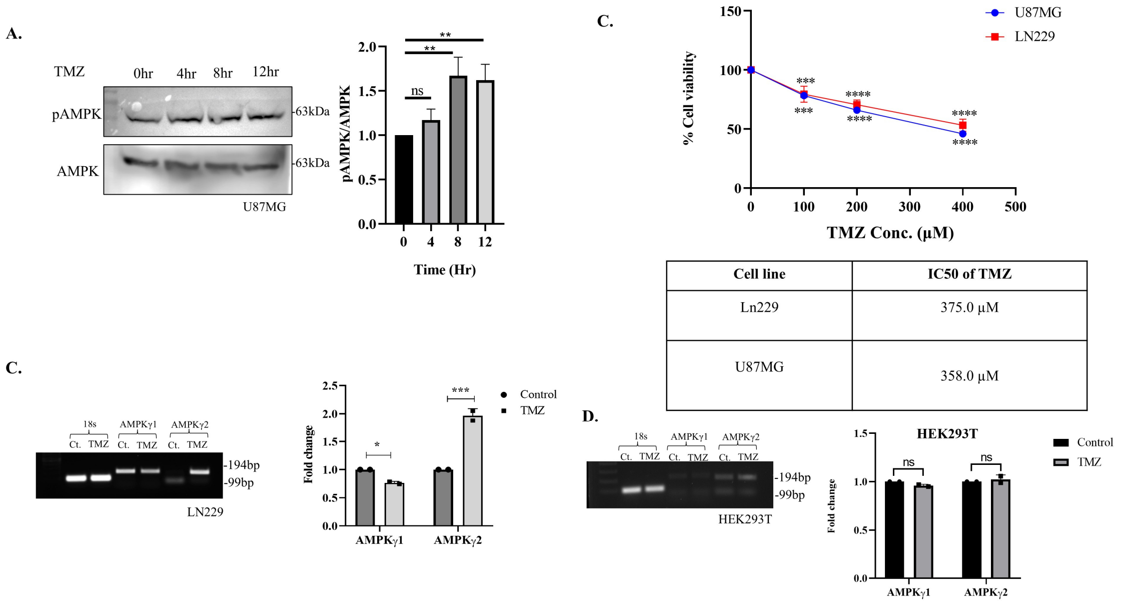

Cell line	IC50 of TMZ
Ln229	375.0 μM
U87MG	358.0 μM

Figure S2: (A) The IC50 of TMZ (μM) in U87MG (358μM), blue line and LN229 (375μM) red line, is represented in a line graph, *y*-axis represents cell survivability. (B) Represents AMPKγ1 and AMPKγ2 expression in control and TMZ-treated LN229 cells. (C) shows pAMPK vs AMPK treated with TMZ at different times in U87MG to check AMPK activation. (D) qPCR of HEK293T cell showing no change in AMPKγ expression while treated with TMZ.

A.

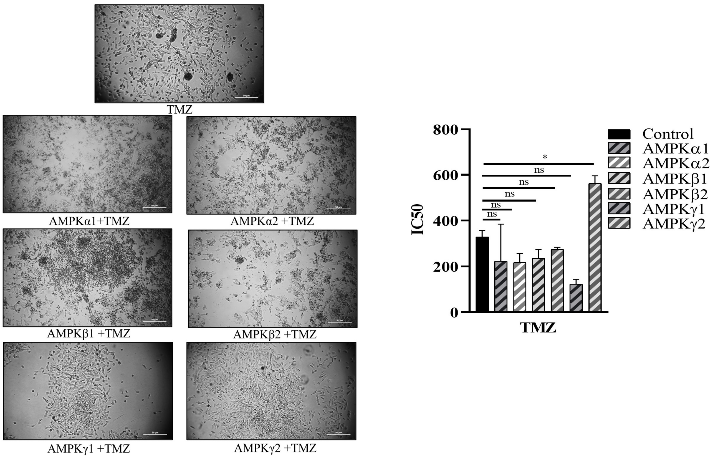

Figure S3: (A) Representing phase contrast images of LN229 cells treated with TMZ along with overexpression of AMPK subunits.

A.

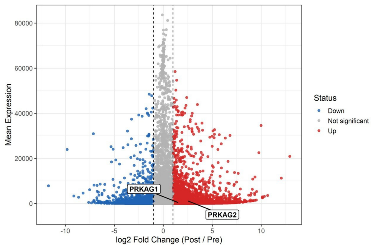

GeneName	Expression_pre	Expression_post	log2FC	meanExpr	Status
PRKAG1	233.9502	672.0879	1.51843993	453.01905	Up
PRKAG2	320.4274	1859.459	2.53309378	1089.9432	Up
PRKAG3	3.202204	2.380245	-0.31401838	2.7912245	Not significan

B.

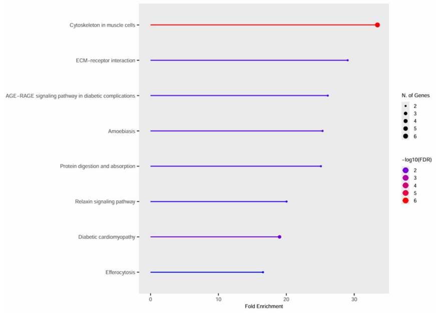

C.

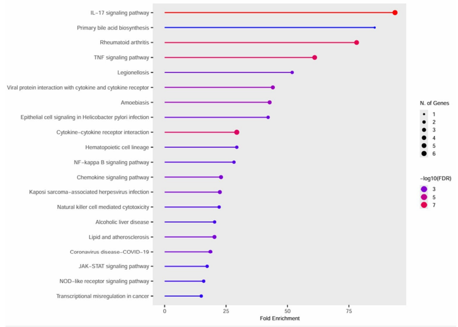

Figure S4: (A) The differential expression of AMPKγ subunits obtained from the transcriptomic dataset (GSE158772). (B-C) Represents pathway enrichment analysis of transcriptomic data. It represents the top 20 pathways that are significantly up- or downregulated. The size of the dot represents the number of genes, and the color represents -log10(FDR).

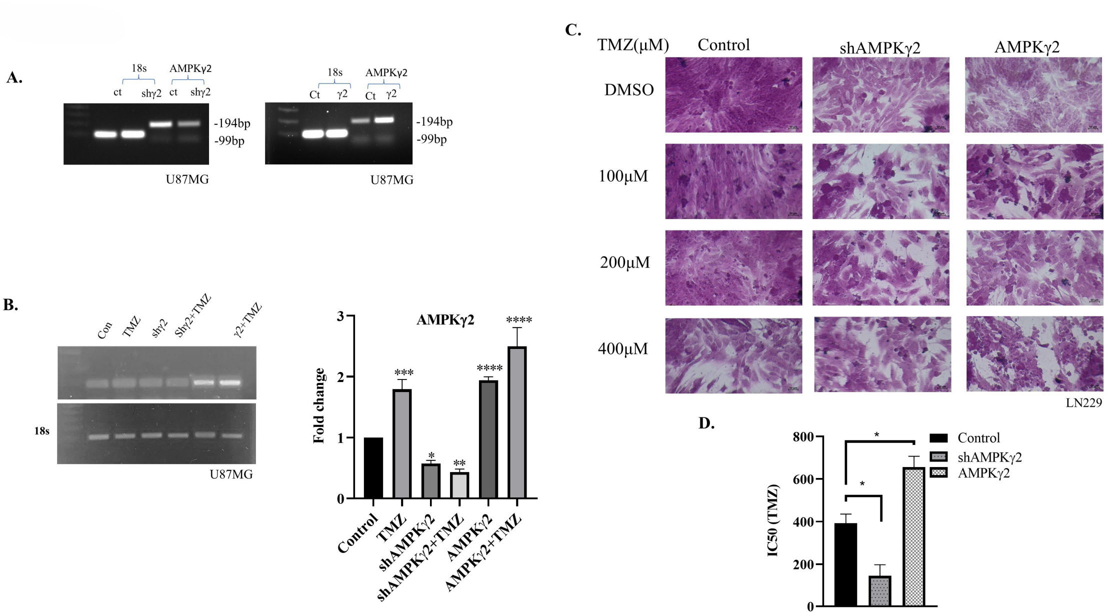

Figure S5: (A) RT-PCR validation of AMPKγ2 knockdown (shAMPKγ2) and AMPKγ2 overexpression in U87MG cells. Expression levels were normalized to 18s rRNA. (B) Quantification of AMPKγ2 expression under control, TMZ treatment, AMPKγ2 knockdown and, overexpression, AMPKγ2 knockdown and overexpression with TMZ treatment conditions, demonstrating efficient modulation of γ2 expression in U87MG cells. (C) Representative crystal violet staining images of LN229 cells under control, AMPKγ2 knockdown (shAMPKγ2), and AMPKγ2 overexpression conditions following treatment with increasing concentrations of TMZ. (B) MTT quantification of TMZ IC50 values in LN229 cells across the indicated conditions. AMPKγ2 overexpression significantly increases the IC50, whereas AMPKγ2 knockdown lowers it. Data are presented as mean ± SEM, and statistical significance is indicated (* $p < 0.05$).

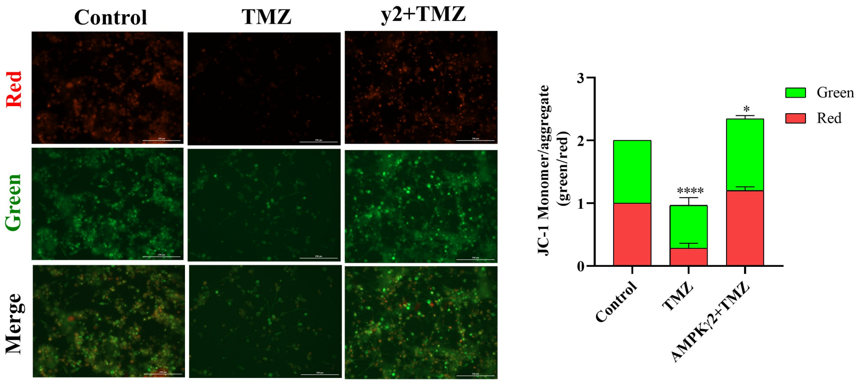

Figure S6: JC-1 staining images showing mitochondrial membrane potential in U87MG cells under control, TMZ-treated, and AMPKγ2 overexpression with TMZ conditions. Red fluorescence indicates polarized (healthy) mitochondria, whereas green fluorescence indicates depolarized mitochondria. TMZ treatment markedly increases green and reduces red fluorescence, indicating mitochondrial depolarization. In contrast, AMPKγ2 overexpression partially restores mitochondrial membrane potential.

Molecular Docking of ATP with Gamma1 subunit of AMPK

CurPocket ID	Vina score	Cavity volume (Å^3)	Center (x, y, z)	Docking size (x, y, z)
C1	-9.1	4111	20, -38, 13	33, 27, 35
C3	-6.4	205	21, -49, 6	21, 21, 21
C5	-6.1	124	33, -52, 13	21, 21, 21
C2	-5.5	534	39, -48, 21	21, 21, 21
C4	-5.5	155	6, -47, 27	21, 21, 21

Molecular Docking of ATP with Gamma2 subunit of AMPK

CurPocket ID	Vina score	Cavity volume (Å^3)	Center (x, y, z)	Docking size (x, y, z)
C1	-7.6	7422	71, -68, 47	28, 35, 35
C4	-5.8	157	66, -72, 29	21, 21, 21
C2	-5.6	315	54, -54, 52	21, 21, 21
C5	-5.3	141	89, -60, 57	21, 21, 21
C3	-5.0	188	77, -66, 73	21, 21, 21

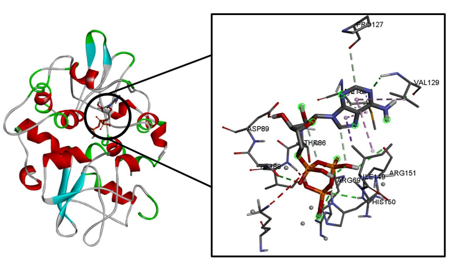

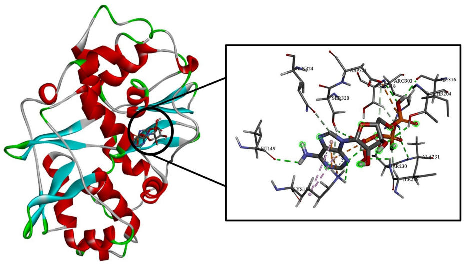

Figure S7: Molecular docking of ATP with AMPKγ1 and γ2 subunits showing binding affinities, cavity volumes, and docking parameters. The docking result for the γ1 subunit indicates the highest binding affinity at cavity C1, with a Vina score of -9.1 kcal/mol. The docking result for the γ2 subunit shows -7.6 kcal/mol but a larger cavity volume.

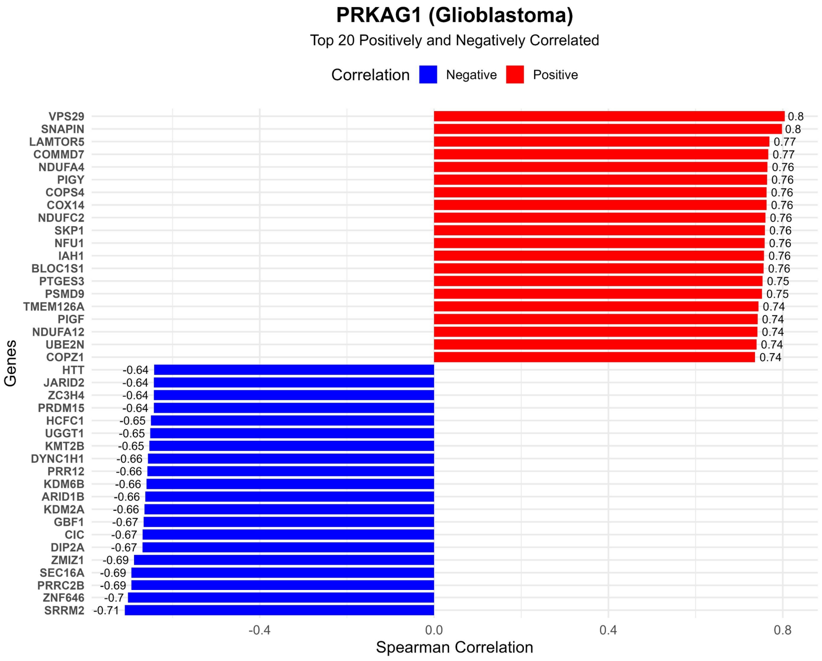

Figure S8: Top 20 positively and negatively correlated genes to *PRKAG1* (AMPKγ1) expression in glioblastoma. Spearman correlation analysis was performed to identify genes associated with *PRKAG1* expression. Positively correlated genes are shown in red bars and negatively correlated genes in blue bars. Correlation coefficients are shown for each gene, with positive correlations ranging from 0.74 to 0.80 and negative correlations ranging from −0.64 to −0.71.

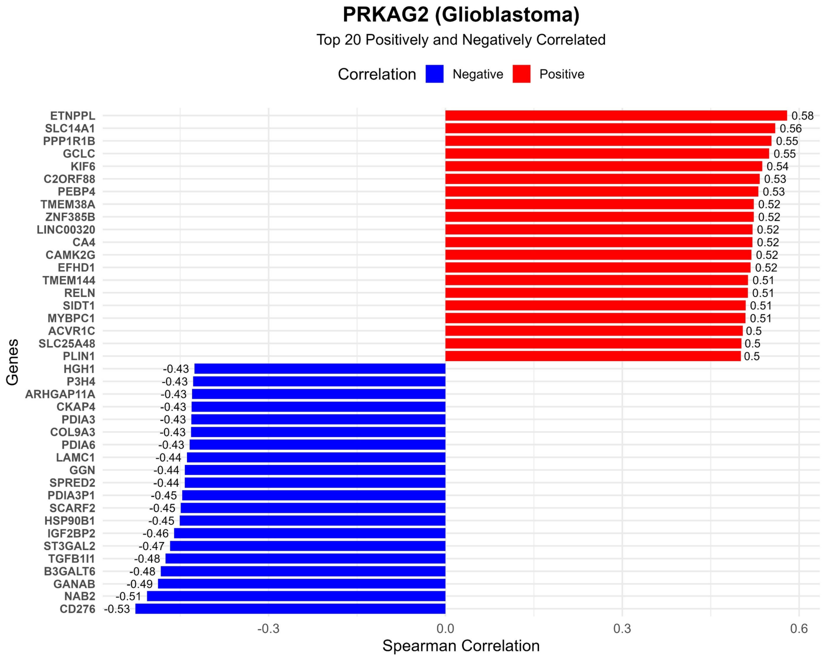

Figure S9: Top 20 positively and negatively correlated genes to *PRKAG2* (AMPKγ2) expression in glioblastoma. Spearman correlation analysis was performed to identify genes associated with *PRKAG1* expression. Positively correlated genes are shown in red bars and negatively correlated genes in blue bars. Correlation coefficients are shown for each gene, with positive correlations ranging from 0.5 to 0.58 and negative correlations ranging from −0.43 to −0.53.

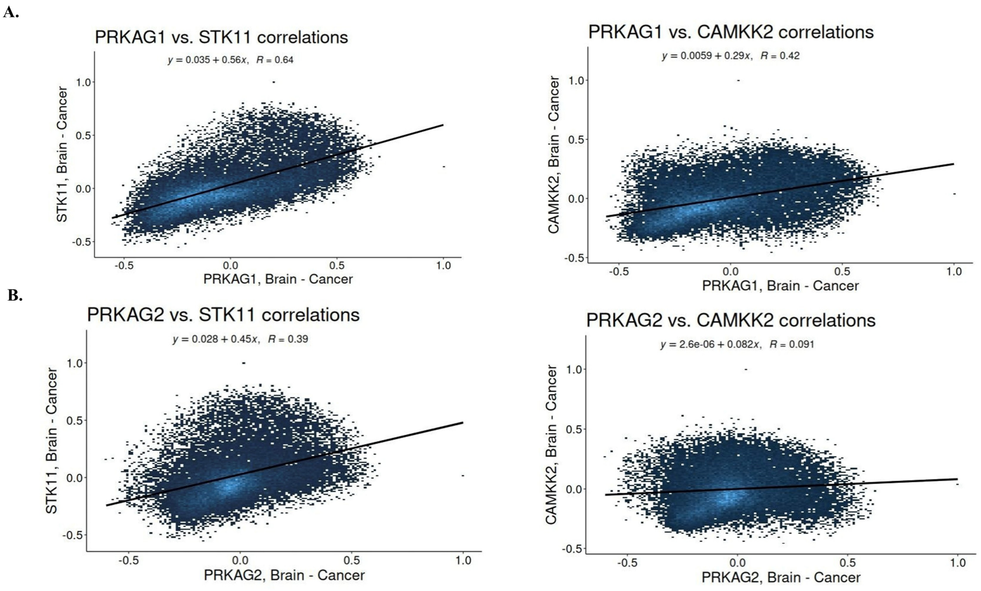

Figure S10: (A) Correlation analysis of *PRKAG1* vs. *SKT11* (R = 0.64) and *CAMKK2* (R = 0.42) shows positive correlation whereas with *PRAKG2* (B) *SKT1* (R = 0.39) showing weak positive correlation and with no correlation with *CAMKK2* (0.091).
